# Supplementary material for: Single atom engineering for radiotherapy-activated immune agonist prodrugs
Source: Nat Commun. 2025 Jul 1;16:6021. doi: 10.1038/s41467-025-60768-4 (PMC12218326; doi:10.1038/s41467-025-60768-4)
Supplement: Supplementary file 1 — Supplementary Information [file 41467_2025_60768_MOESM1_ESM.pdf]

Supplementary Information for

Single Atom Engineering for Radiotherapy-Activated Immune Agonist Prodrugs

Zexuan Ding[1,#], Xiaozhe Yin[2,#], Yuedan Zheng[3,#], Yiyan Li[4,#], Huanhuan Ge[1], Jianshu Feng[1], Ziyang Wang[3], Simiao Qiao[1], Qi Sun[4], Fashuo Yu[1], Zhanshan Hou[1], Yang-Xin Fu[1,2,*], Zhibo Liu[1,3,4,*]

[1]Changping Laboratory, Beijing 102206, China

[2]Department of Basic Medical Sciences, School of Medicine, Tsinghua University, Beijing 100084, China

[3]Beijing National Laboratory for Molecular Sciences, Radiochemistry and Radiation Chemistry Key Laboratory of Fundamental Science, Key Laboratory of Bioorganic Chemistry and Molecular Engineering of Ministry of Education, College of Chemistry and Molecular Engineering, Peking University, Beijing 100871, China

[4]Peking University-Tsinghua University Centre for Life Sciences, Peking University, Beijing 100871, China

[#]These authors contributed equally to this work.

[*]To whom correspondence should be addressed.

E-mail Y.F (yangxinfu@tsinghua.edu.cn) and Z.L (zbliu@pku.edu.cn).

(1) Synthetic routes of oxygen-engineered agonists *O*-R848, *O*-852A, *O*-CL075, *O*-BBIQ, and *O*-IMQ4.

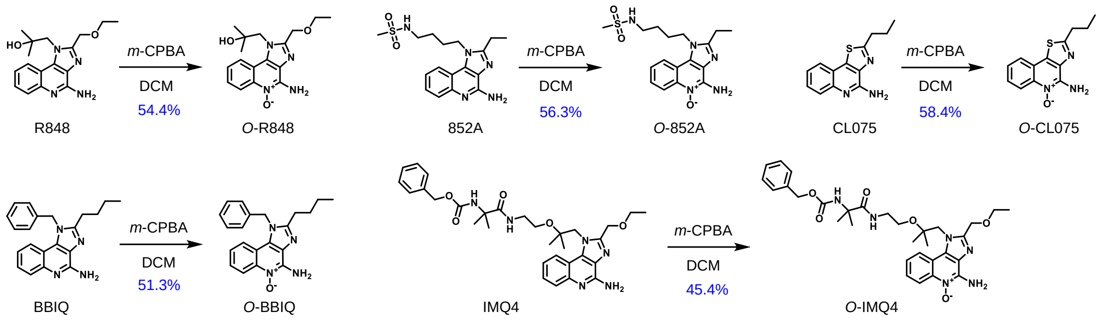

(2) Synthetic routes of oxygen-engineered agonists *O*-T785, *O*-Gar, *O*-IMQ1, *O*-IMQ2, and *O*-IMQ3

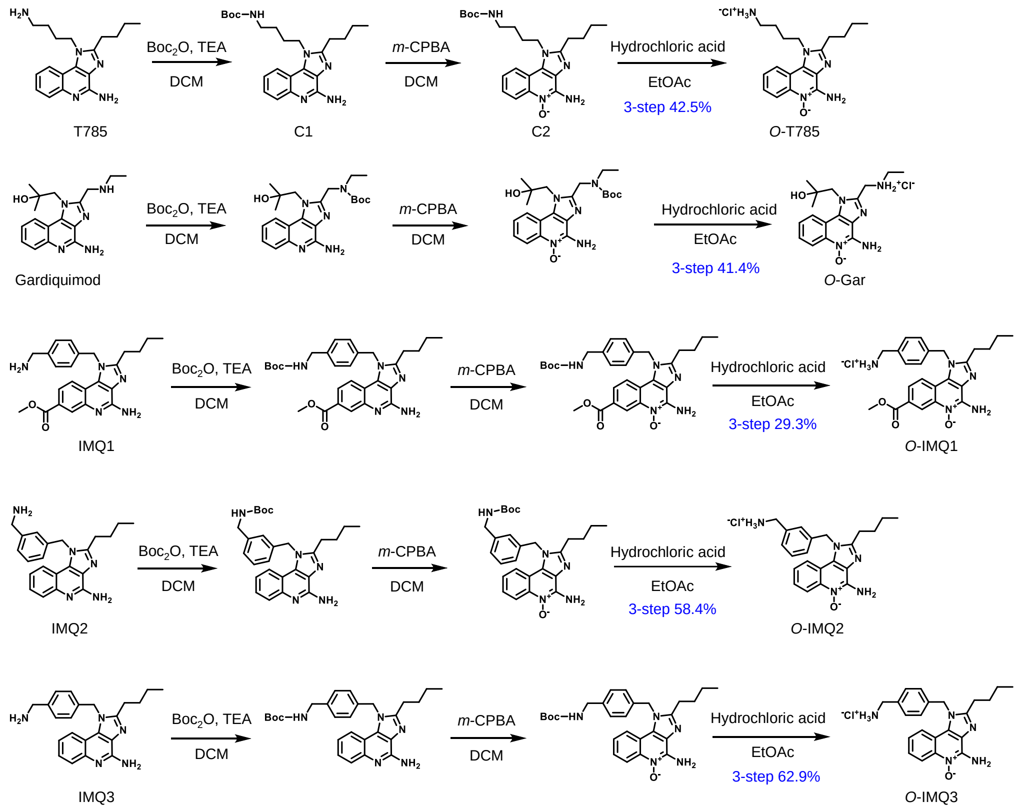

(3) Synthetic routes of oxygen-engineered agonist *O*-CL097

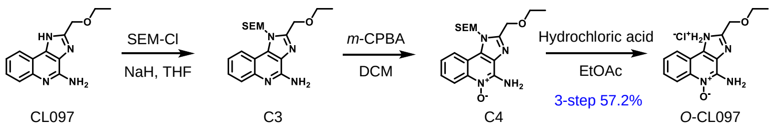

Supplementary Fig. 1. Synthetic routes of oxygen atom-engineered immune agonist prodrugs. The oxygen atom-engineered immune agonist prodrugs were synthesized by three general methods: (1) One-step oxidation; (2) *N*-oxidation after amine protection via Boc group; (3) *N*-oxidation after amine protection via SEM group.

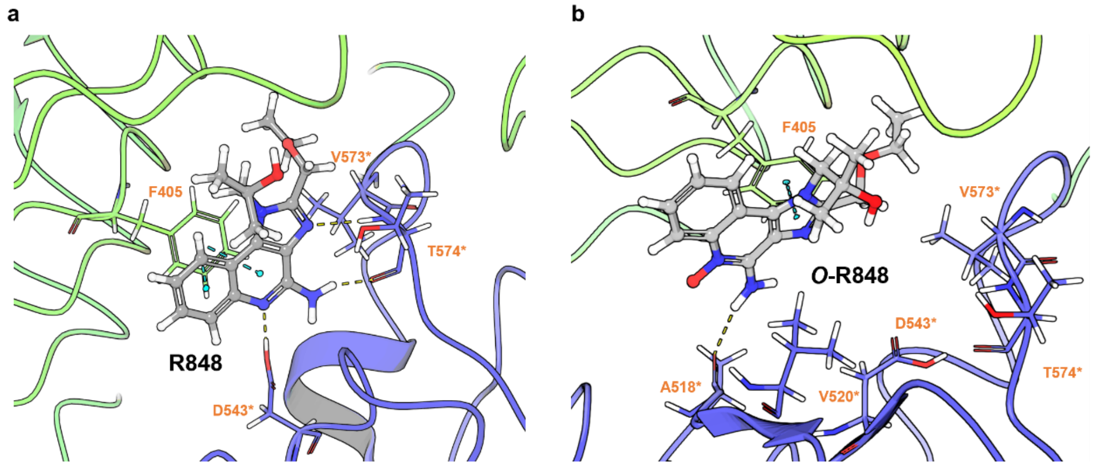

Supplementary Fig. 2. Representative snapshots of agonists-TLR8. a,b R848-TLR8 (**a**) and *O*-R848-TLR8 (**b**) complex in equilibrium during 30 ns MD simulations.

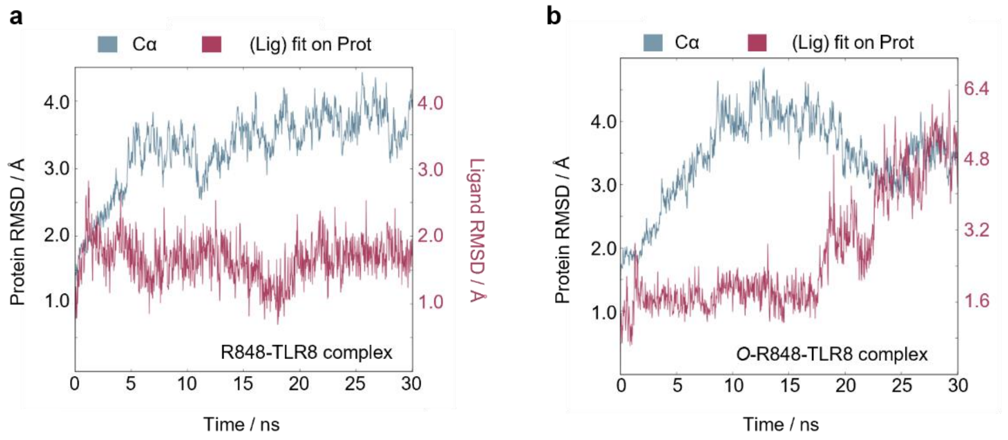

Supplementary Fig. 3. Protein RMSD of agonists-TLR8. a,**b**, R848-TLR8 (**a**) and *O*-R848-TLR8 (**b**) complex in equilibrium during 30 ns MD simulations.

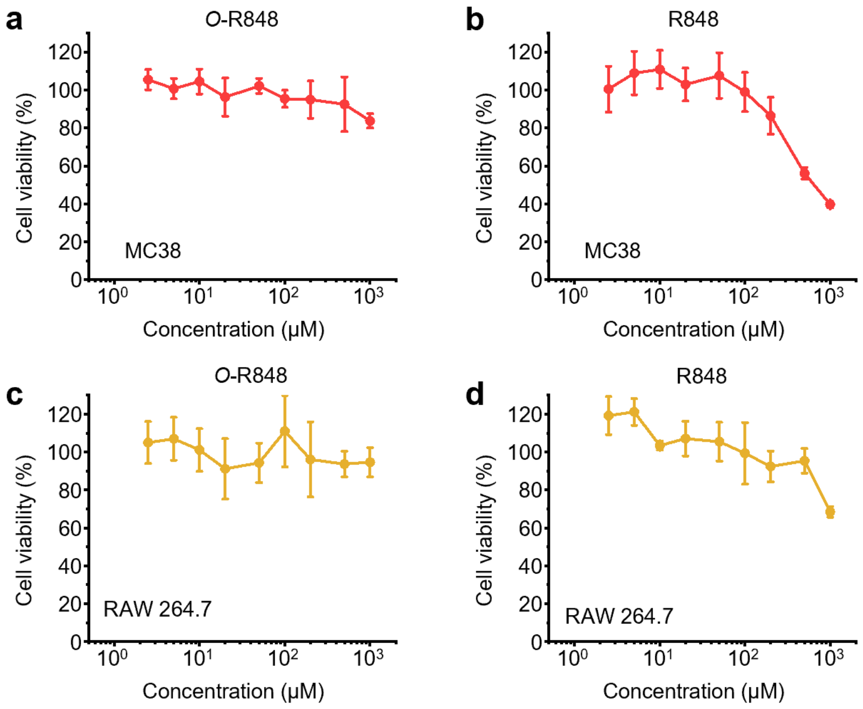

Supplementary Fig. 4. Cytotoxicity of *O*-R848 and R848. a-d, Cell viability of MC38 incubated with (**a**) *O*-R848 and (**b**) R848 for 24 h, and cell viability of RAW264.7 incubated with (**c**) *O*-R848 and (**d**) R848 for 24 h. Data are presented as mean values ± s.d., n = 6 independently tested cell samples for each group. Source data are provided as a Source Data file.

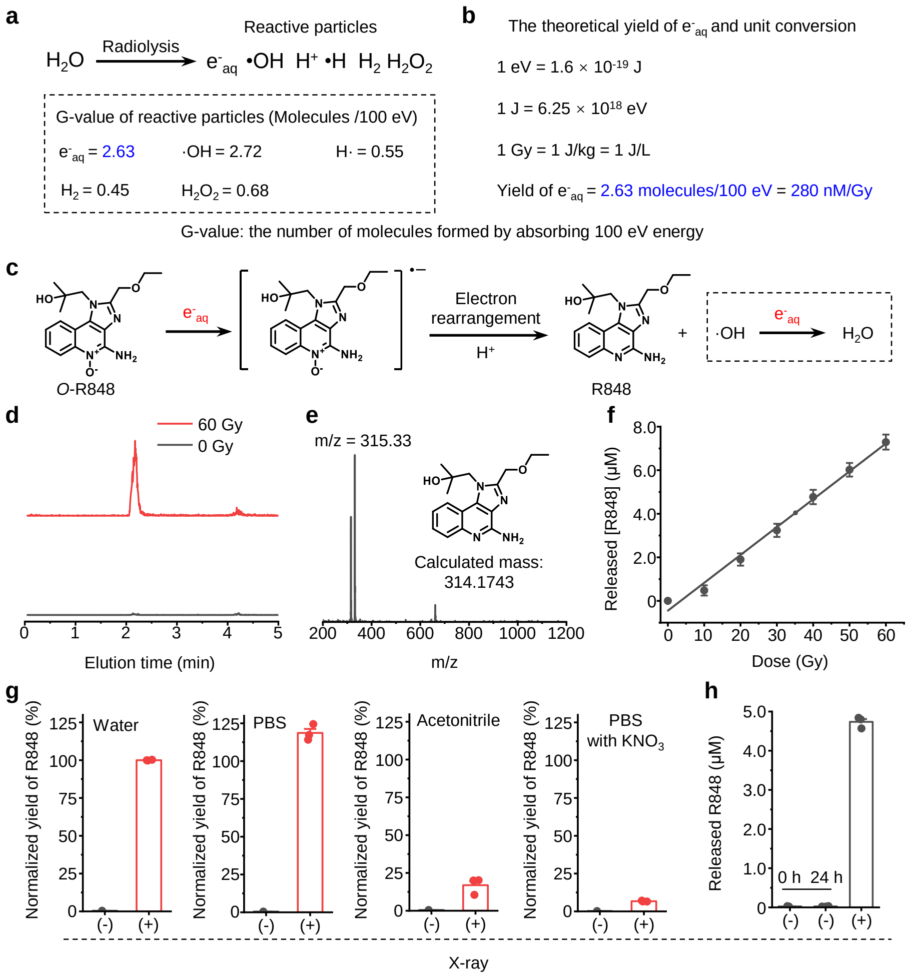

Supplementary Fig. 5. X-ray-induced reduction of *O*-R848 via e^-_{aq} generated from water radiolysis. **a**, Water radiolysis generates various reactive particles with different G-values. G-value means the number of molecules formed by absorbing 100 eV energy in the system. **b**, The theoretical yield of e^-_{aq} and description of unit conversion. **c**, Proposed mechanism of the e^-_{aq}-mediated reduction of *O*-R848. **d**, UPLC-MS spectra of generated R848 from *O*-R848 (10 μM) in PBS. **e**, Positive ion mode mass spectrum is shown for released R848. **f**, Radiation dosage-dependent releasing of R848 from *O*-R848 (10 μM in PBS) determined by UPLC-MS. **g**, Normalized amount of generated R848 from *O*-R848 (10 μM in pure water, PBS, acetonitrile, and PBS with e_{aq}^- quencher KNO_3, respectively) with and without 60 Gy irradiation determined by UPLC-MS. **h**, *O*-R848 (10 μM) was incubated with living MC38 cells for 24 h or irradiated 60 Gy with living MC38 cells. The yield of released R848 was determined by UPLC-MS. In **f**-**h**, *n* = 3 independently tested samples for each group. Data are presented as mean values ± s.d.. Source data are provided as a Source Data file.

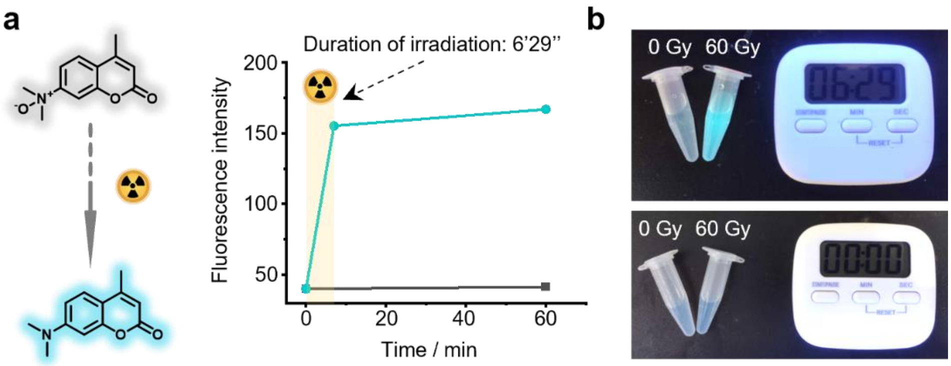

Supplementary Fig. 6. Instant deoxy reaction by X-ray. a,**b**, Time-dependent fluorescence intensity change (**a**) and photographs (**b**) of *O*-coumarin after 60 Gy radiation. n = 3 independently tested samples for each group. Data are presented as mean values ± s.d.. Source data are provided as a Source Data file.

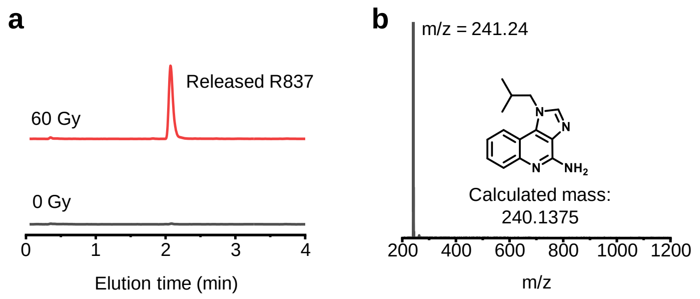

Supplementary Fig. 7. X-ray-induced R837 releasing from *O*-R837 (10 μM) in PBS determined by UPLC-MS. a, UPLC-MS spectra of generated R837. **b**, Positive ion mode mass spectrum is shown for released R837. Source data are provided as a Source Data file.

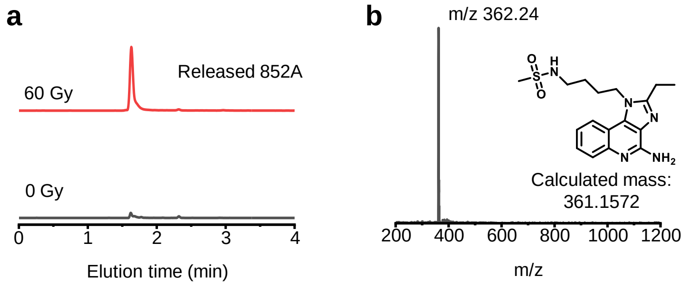

Supplementary Fig. 8. X-ray-induced 852A releasing from *O*-R852A (10 μM) in PBS determined by UPLC-MS. a, UPLC-MS spectra of generated 852A. **b**, Positive ion mode mass spectrum is shown for released 852A. Source data are provided as a Source Data file.

.

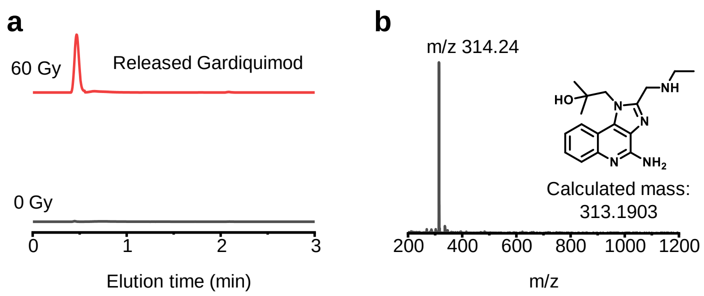

Supplementary Fig. 9. X-ray-induced Gardiquimod releasing from *O*-Gar (10 µM) in PBS determined by UPLC-MS. a, UPLC-MS spectra of generated Gardiquimod. **b**, Positive ion mode mass spectrum is shown for released Gardiquimod. Source data are provided as a Source Data file.

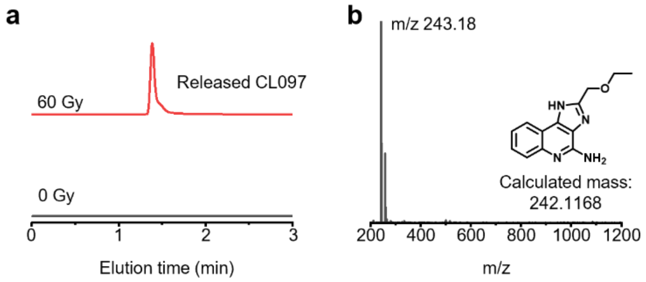

Supplementary Fig. 10. X-ray-induced CL097 releasing from *O*-CL097 (10 µM) in PBS determined by UPLC-MS. a, UPLC-MS spectra of generated CL097. **b**, Positive ion mode mass spectrum is shown for released CL097. Source data are provided as a Source Data file.

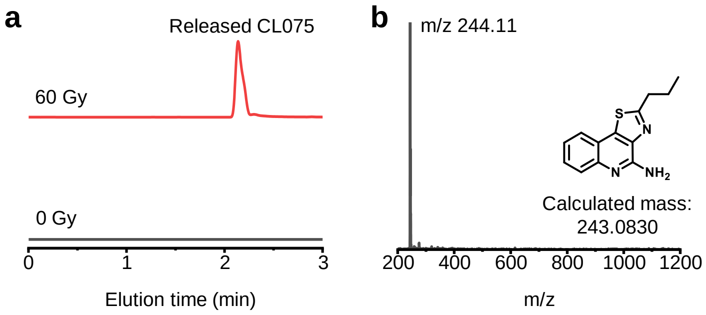

Supplementary Fig. 11. X-ray-induced CL075 releasing from *O*-CL075 (10 μM) in PBS determined by UPLC-MS. a, UPLC-MS spectra of generated CL075. **b**, Positive ion mode mass spectrum is shown for released CL075. Source data are provided as a Source Data file.

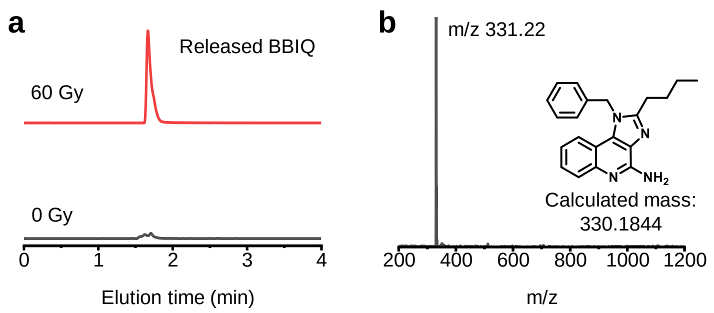

Supplementary Fig. 12. X-ray-induced BBIQ releasing from *O*-BBIQ (10 µM) in PBS determined by UPLC-MS. a, UPLC-MS spectra of generated BBIQ. **b**, Positive ion mode mass spectrum is shown for released BBIQ. Source data are provided as a Source Data file.

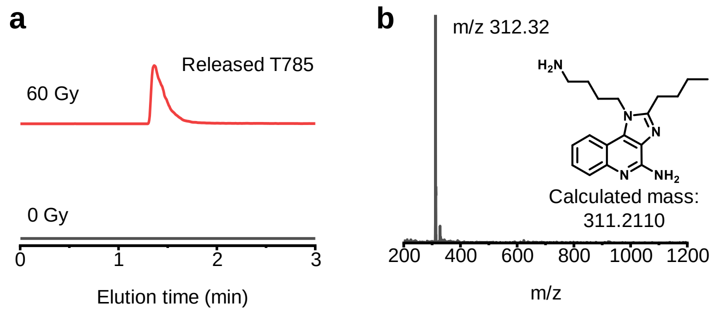

Supplementary Fig. 13. X-ray-induced T785 releasing from *O*-T785 (10 μM) in PBS determined by UPLC-MS. a, UPLC-MS spectra of generated T785. **b**, Positive ion mode mass spectrum is shown for released T785. Source data are provided as a Source Data file.

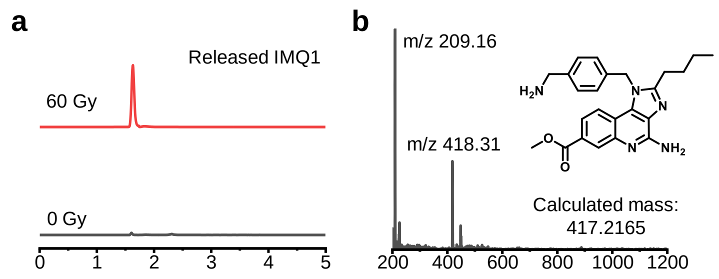

Supplementary Fig. 14. X-ray-induced IMQ1 releasing from *O*-IMQ1 (10 µM) in PBS determined by UPLC-MS. a, UPLC-MS spectra of generated IMQ1. **b**, Positive ion mode mass spectrum is shown for released IMQ1. Source data are provided as a Source Data file.

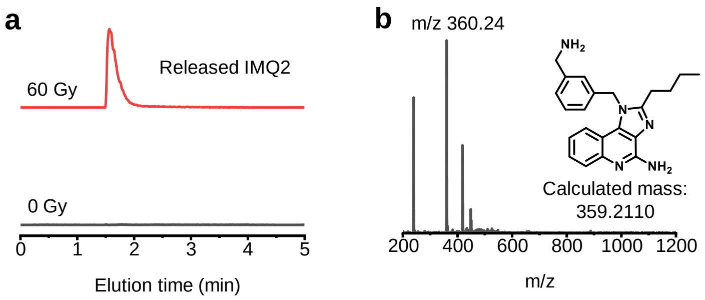

Supplementary Fig. 15. X-ray-induced IMQ2 releasing from *O*-IMQ2 (10 μM) in PBS determined by UPLC-MS. a, UPLC-MS spectra of generated IMQ2. **b**, Positive ion mode mass spectrum is shown for released IMQ2. Source data are provided as a Source Data file.

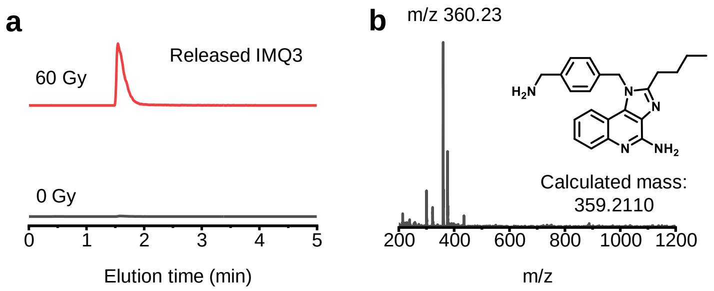

Supplementary Fig. 16. X-ray-induced IMQ3 releasing from *O*-IMQ3 (10 μM) in PBS determined by UPLC-MS. a, UPLC-MS spectra of generated IMQ3. **b**, Positive ion mode mass spectrum is shown for released IMQ3. Source data are provided as a Source Data file.

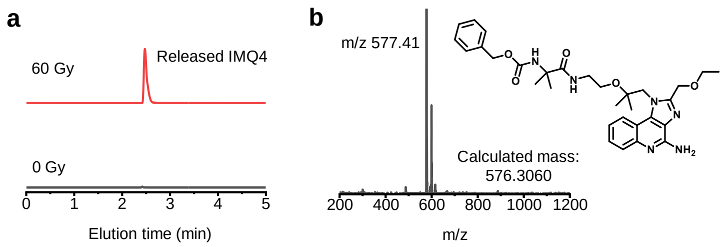

Supplementary Fig. 17. X-ray-induced IMQ4 releasing from *O*-IMQ4 (10 μM) in PBS determined by UPLC-MS. a, UPLC-MS spectra of generated IMQ4. **b**, Positive ion mode mass spectrum is shown for released IMQ4. Source data are provided as a Source Data file.

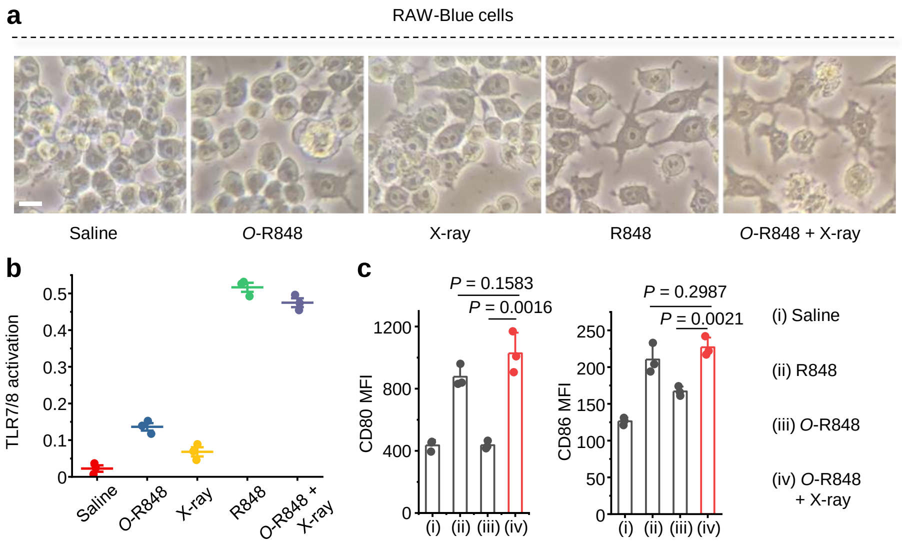

Supplementary Fig. 18. X-ray rescues the immunostimulatory activity of *O*-R848 in APCs. **a**-**c**, Activation of RAW-Blue and RAW264.7 cells by X-ray-activated *O*-R848. Representative photos (**a**) and TLR7/8 activation (**b**) of RAW-Blue cells treated with *O*-R848 (1 μM), X-ray irradiation (10 Gy), *O*-R848 (1 μM) with subsequent X-ray irradiation (10 Gy), and R848 (1 μM) as a positive control. Scale bar in **a**: 10 μm. **c**, CD80/86 expression in RAW264.7. Data are presented as mean values ± s.d., $n = 3$ independently tested samples for each group, two-tailed unpaired Student's *t*-test. Source data are provided as a Source Data file.

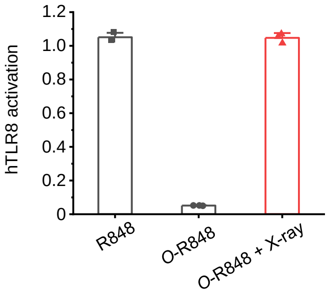

Supplementary Fig. 19. Human TLR8 (hTLR8) activation of R848, *O*-R848 and *O*-R848 treated with X-ray in hTLR8 report cells. Data are presented as mean values ± s.d., *n* = 3 independently tested samples for each group. Source data are provided as a Source Data file.

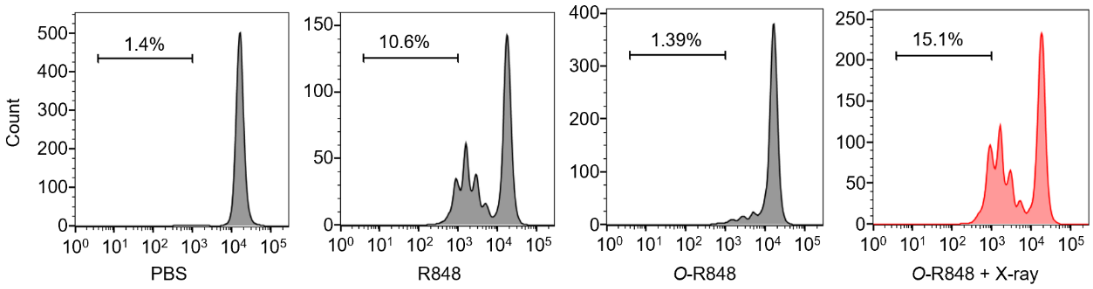

Supplementary Fig. 20. Representative flow cytometric quantitative results of OT I CD8^{+} T cells.

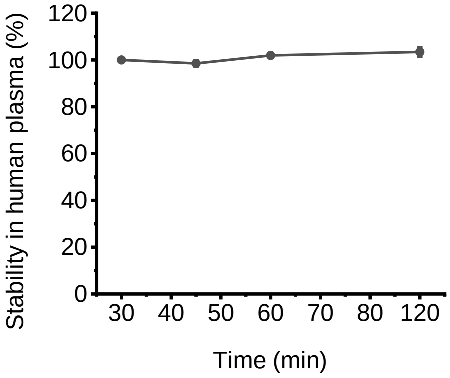

Supplementary Fig. 21. Stability of *O*-R848 in human plasma in 2 h at 37 °C. n = 3 independently tested samples for each group. Data are presented as mean values ± s.d.. Source data are provided as a Source Data file.

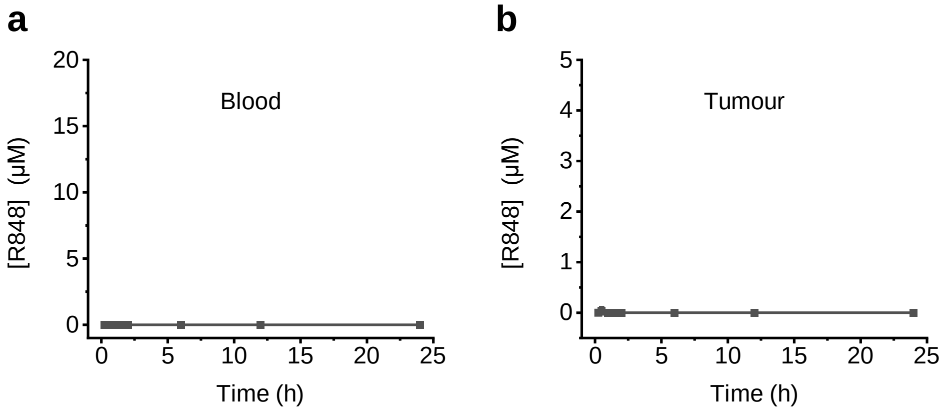

Supplementary Fig. 22. R848 release in blood and tumour without radiotherapy treated. **a,b.** R848 release in blood (**a**) and tumour (**b**) detected by UPLC-MS. All mice were 6-8 weeks old, female, *n* = 5 mice for each group. Data are presented as mean values ± s.d.. Source data are provided as a Source Data file.

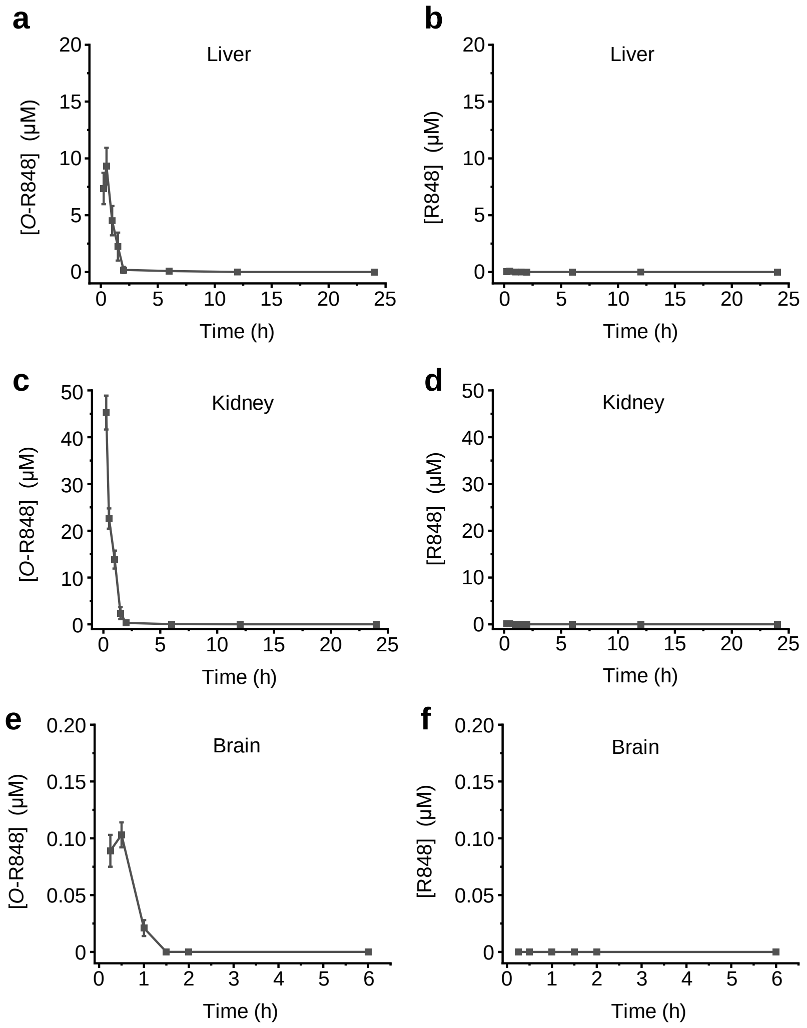

Supplementary Fig. 23. Time-dependent accumulation of *O*-R848 and release of R848 in liver, kidney, and brain tissues without radiotherapy treated. Concentration of *O*-R848 and R848 detected in liver (**a** and **b**), kidney (**c** and **d**), brain (**e** and **f**) by UPLC-MS. All mice were 6-8 weeks old, female, n = 5 mice for each group. Data are presented as mean values ± s.d.. Source data are provided as a Source Data file.

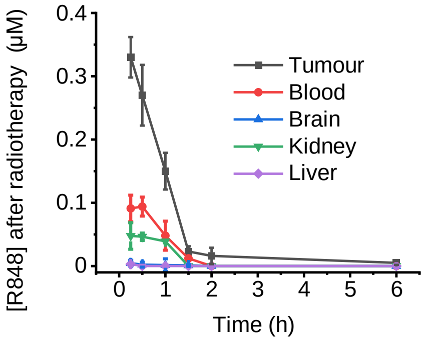

Supplementary Fig. 24. Time-dependent distribution of released R848 after radiotherapy treated, detected by UPLC-MS. All mice were 6-8 weeks old, female, *n* = 5 mice for each group. Data are presented as mean values ± s.d.. Source data are provided as a Source Data file.

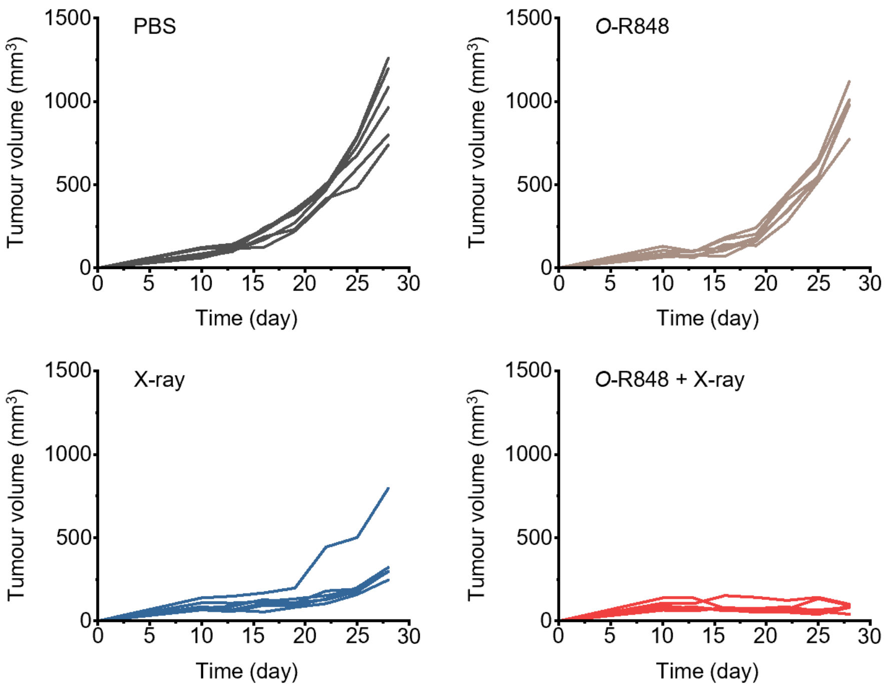

Supplementary Fig. 25. Tumour volume of an individual mouse after treatment for 4T1 bearing BALB/c mice. All mice were 6-8 weeks old, female, n = 6 mice per group. Source data are provided as a Source Data file.

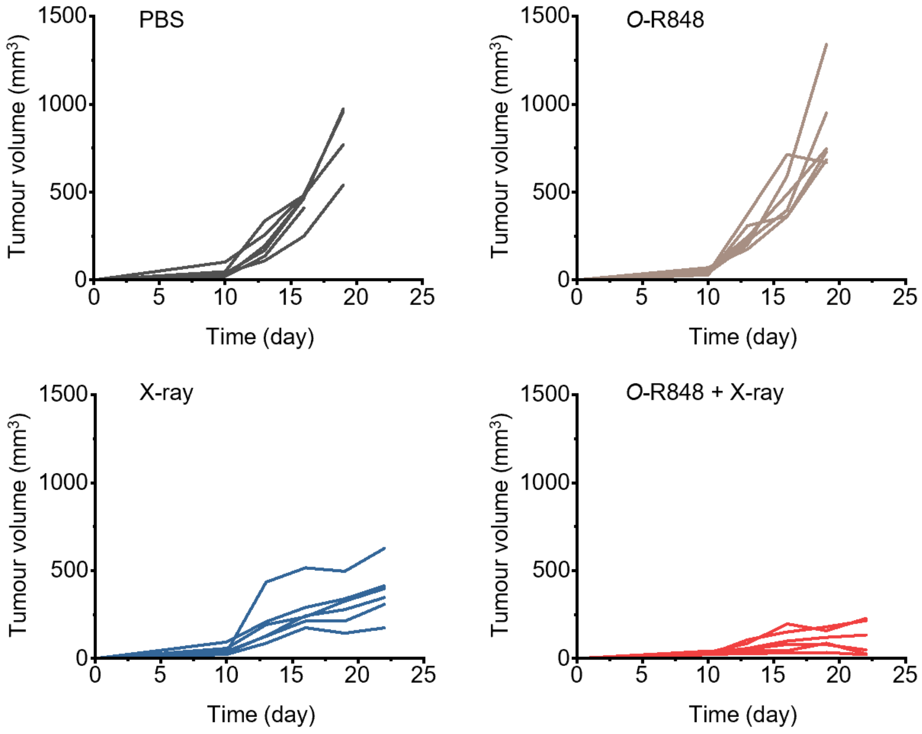

Supplementary Fig. 26. Tumour volume of an individual mouse after treatment for B16 bearing C57BL/6J mice. All mice were 6-8 weeks old, female, n = 6 mice per group. Source data are provided as a Source Data file.

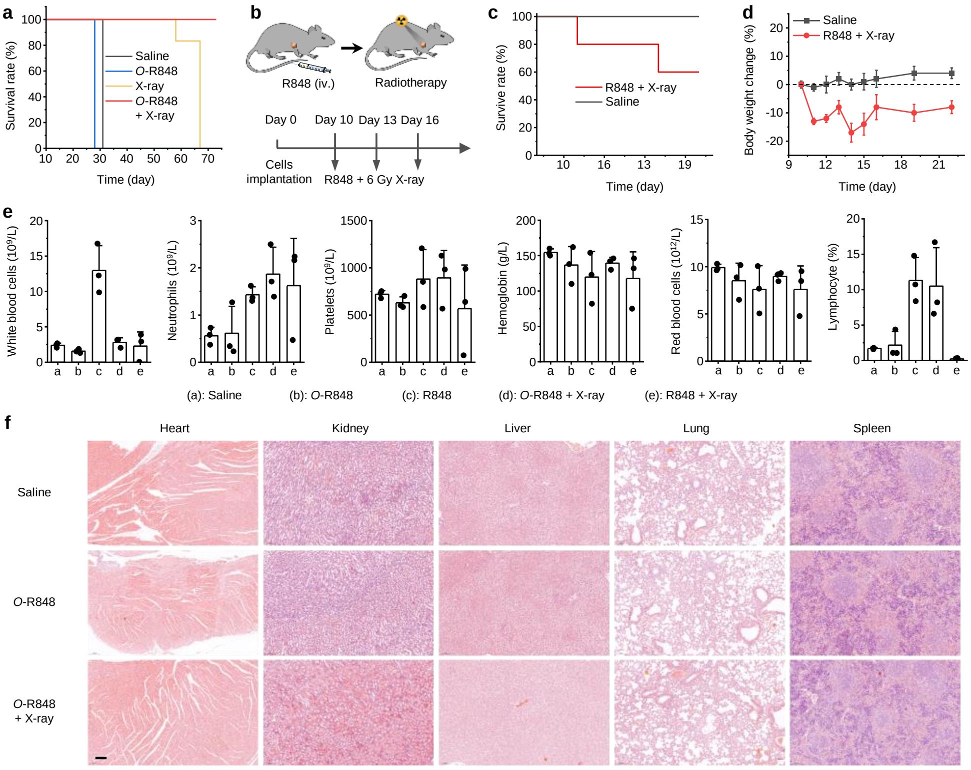

Supplementary Fig. 27. Radiotherapy activates *O*-R848, reducing the side effects of combined radiotherapy and immune agonist treatment. a, Survival rate of tumour-bearing mice post-treatments of *O*-R848 (30 µmol/kg) followed by 6 Gy radiotherapy activation (3 treatments). **b-d**, Treatment with R848 and radiotherapy. All mice were 6-8 weeks old, female, n = 5 mice for each group. **b**, C57BL/6J mice were implanted subcutaneously with MC38 cells on day 0, followed by intravenous administration of R848 (30 µmol/kg) and radiotherapy (6 Gy for each treatment). **c**, Survival rate in 21 days of tumour-bearing mice post-treatments of R848 (30 µmol/kg) followed by 6 Gy radiotherapy activation (3 treatments). **d**, Weight change curves. **e**, Complete blood count analysis. Blood samples (n = 5 mice for each group) were collected at a time point of 7 days post-treatments. **f**, Representative haematoxylin and eosin (H&E) staining from mice with indicated treatments. scale bar = 100 µm. In **a-e**, data are presented as mean values ± s.d.. Source data are provided as a Source Data file.

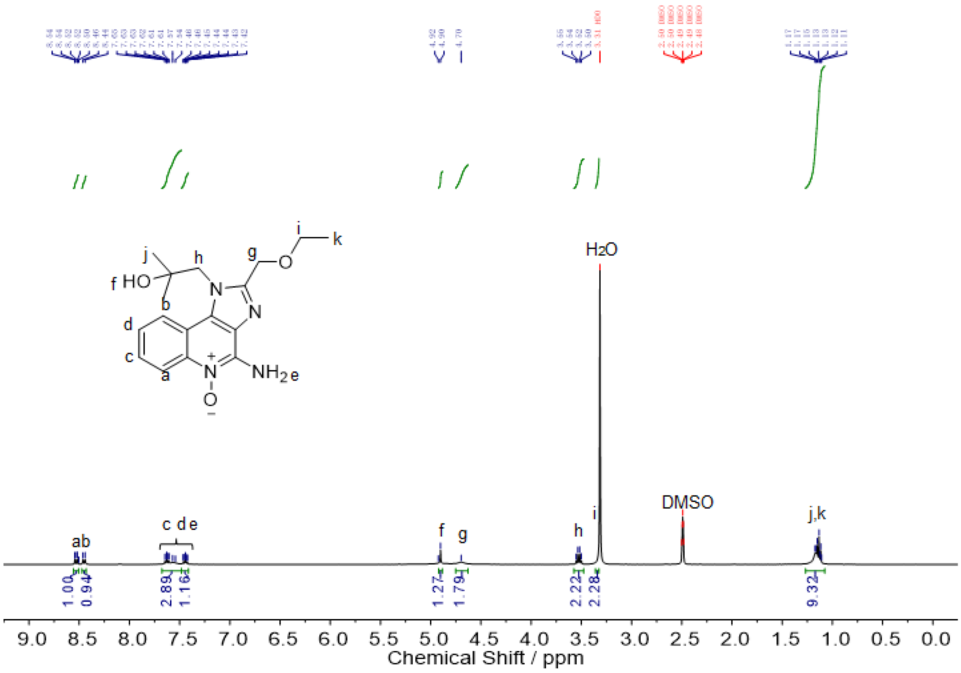

Supplementary Fig. 28. ^{1}H NMR spectrum of *O*-R848.

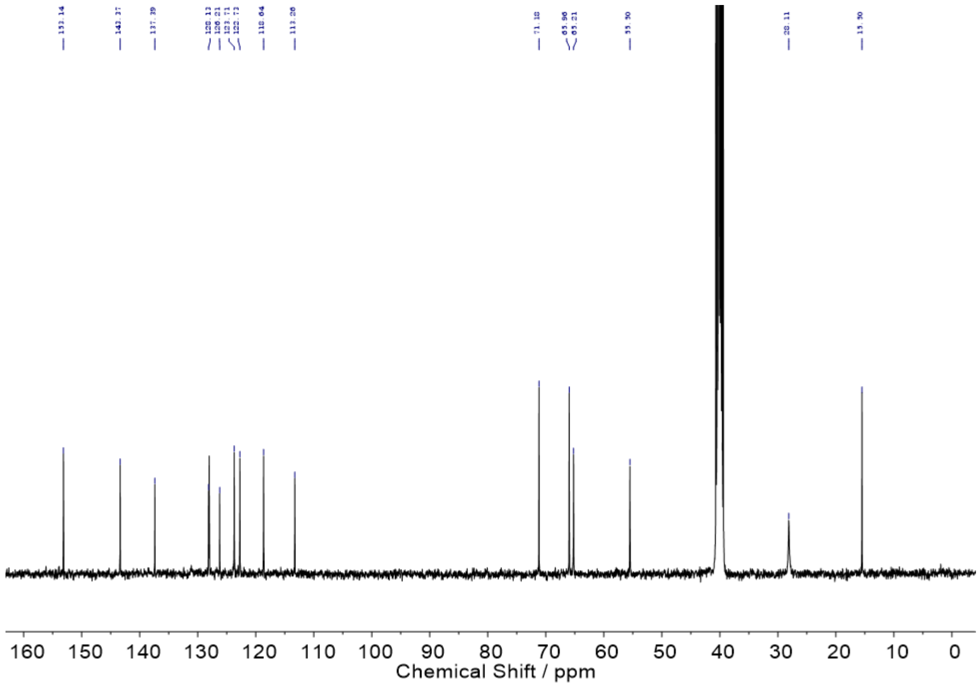

Supplementary Fig. 29. ^{13}C NMR spectrum of *O*-R848.

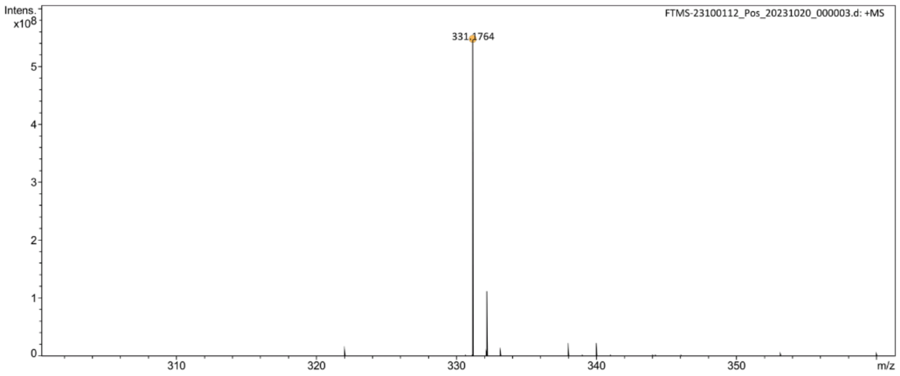

Meas. m/z	#	Ion Formula	Score	m/z	err [ppm]	Mean err [ppm]	mSigma	rdb	e⁻ Conf	N-Rule
331.1764	1	C17H23N4O3	100.00	331.1765	0.2	-0.0	5.1	9.0	even	ok

Supplementary Fig. 30. High-resolution mass spectrum of *O*-R848.

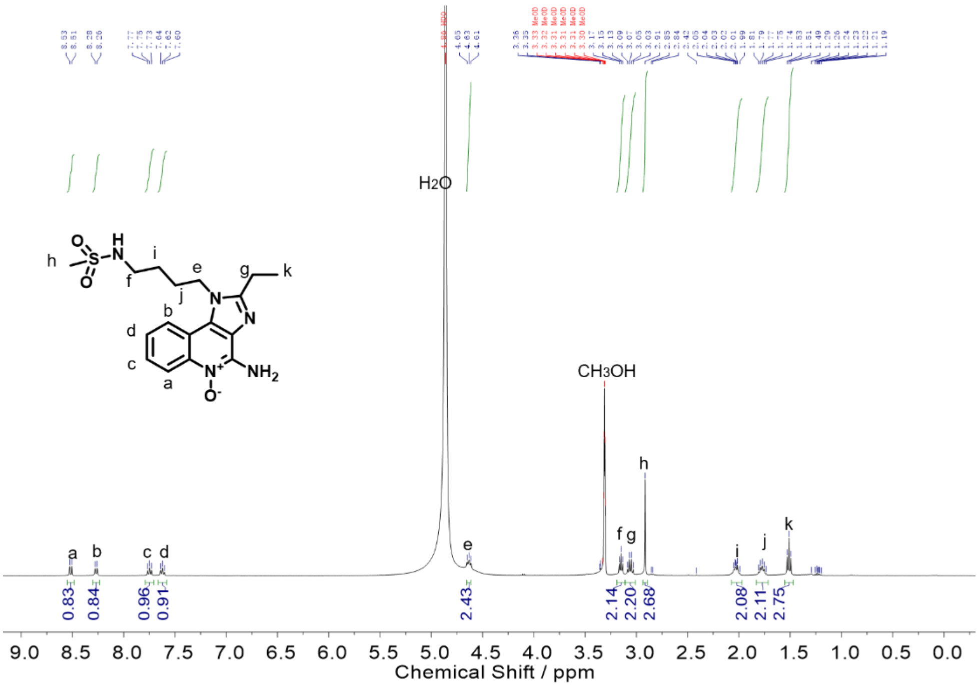

Supplementary Fig. 31. ^{1}H NMR spectrum of *O*-852A.

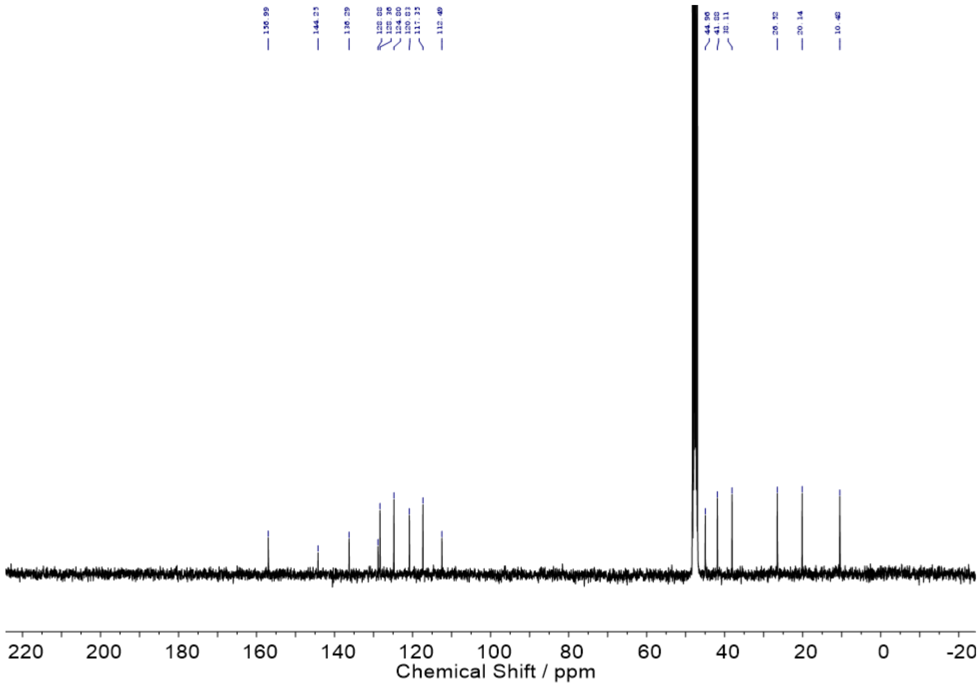

Supplementary Fig. 32. ^{13}C NMR spectrum of *O*-852A.

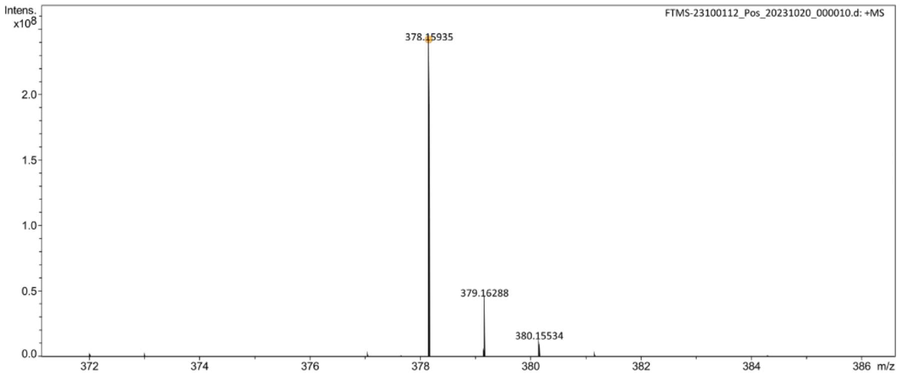

Meas. m/z	#	Ion Formula	Score	m/z	err [ppm]	Mean err [ppm]	mSigma	rdb	e⁻ Conf	N-Rule
378.15935	1	C17H24N5O3S	100.00	378.15944	0.22	-1.25	90.0	11.0	even	ok

Supplementary Fig. 33. High-resolution mass spectrum of *O*-852A.

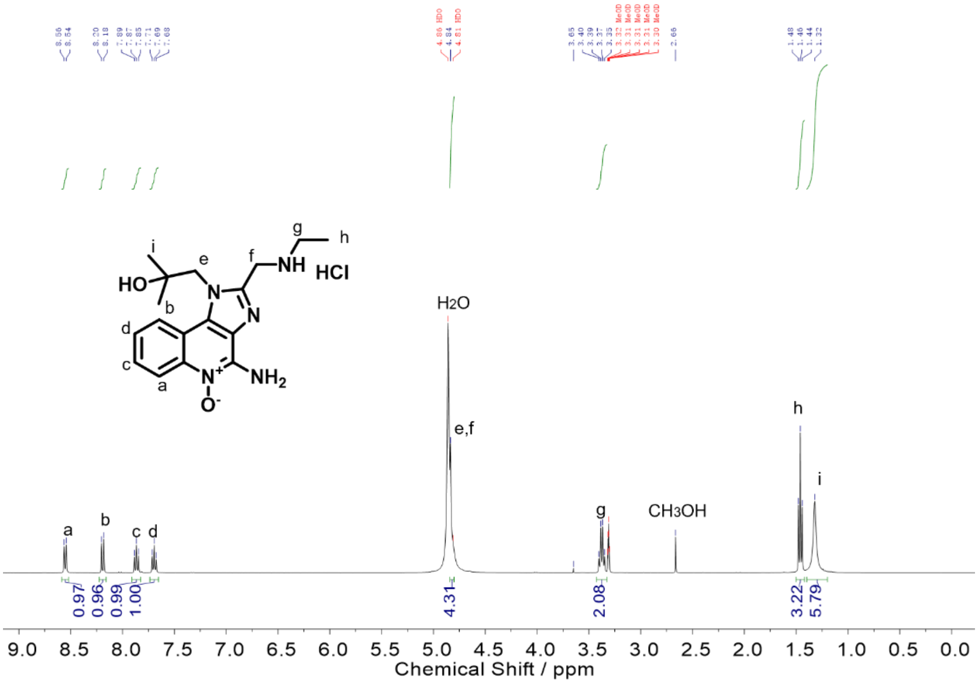

Supplementary Fig. 34. ^{1}H NMR spectrum of *O*-Gardiquimod.

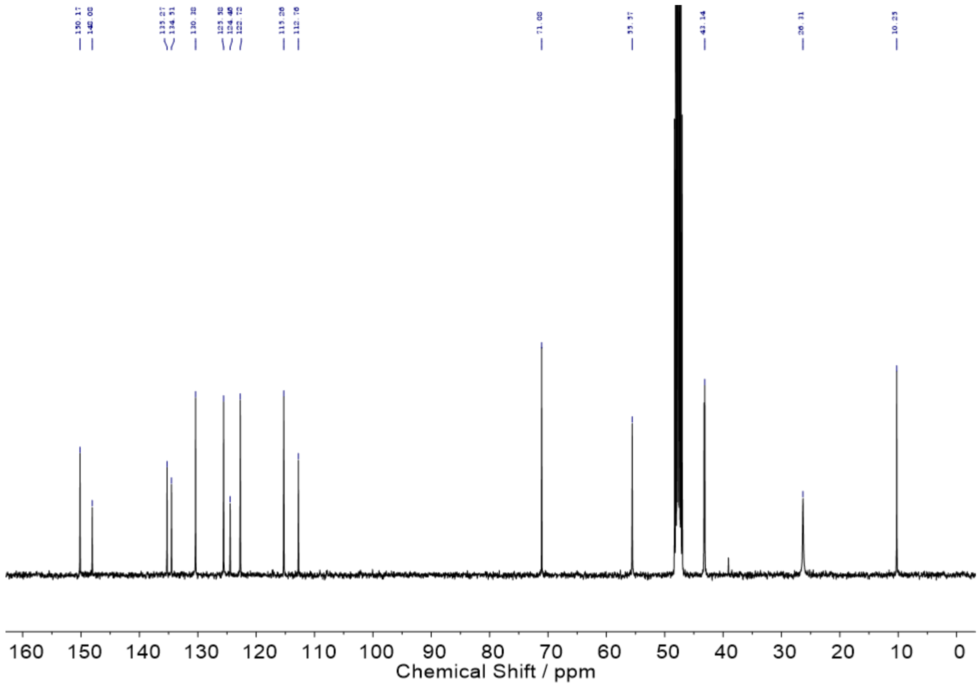

Supplementary Fig. 35. ^{13}C NMR spectrum of *O*-Gardiquimod.

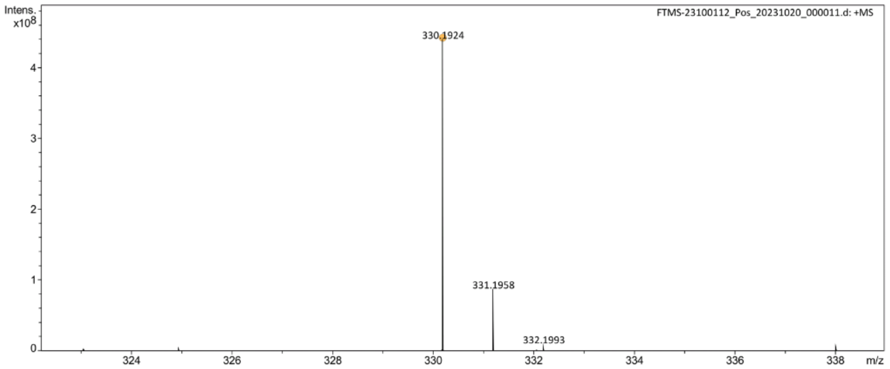

Meas. m/z	#	Ion Formula	Score	m/z	err [ppm]	Mean err [ppm]	mSigma	rdb	e⁻ Conf	N-Rule
330.1924	1	$C_{17}H_{24}N_5O_2$	100.00	330.1925	0.2	-0.1	3.9	9.0	even	ok

Supplementary Fig. 36. High-resolution mass spectrum of *O*-Gardiquimod.

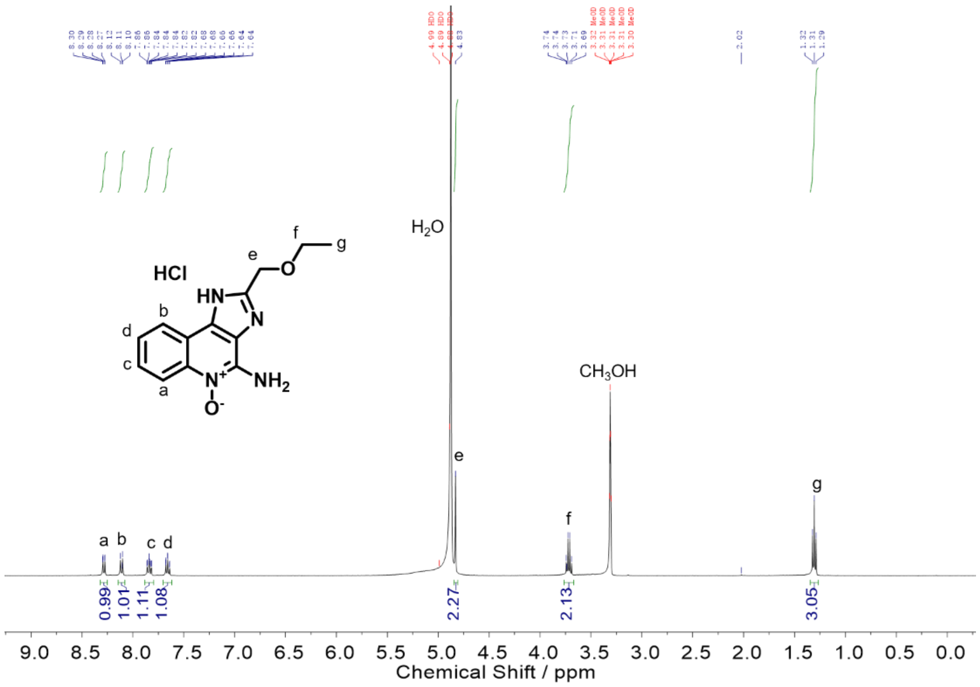

Supplementary Fig. 37. ^{1}H NMR spectrum of *O*-CL097.

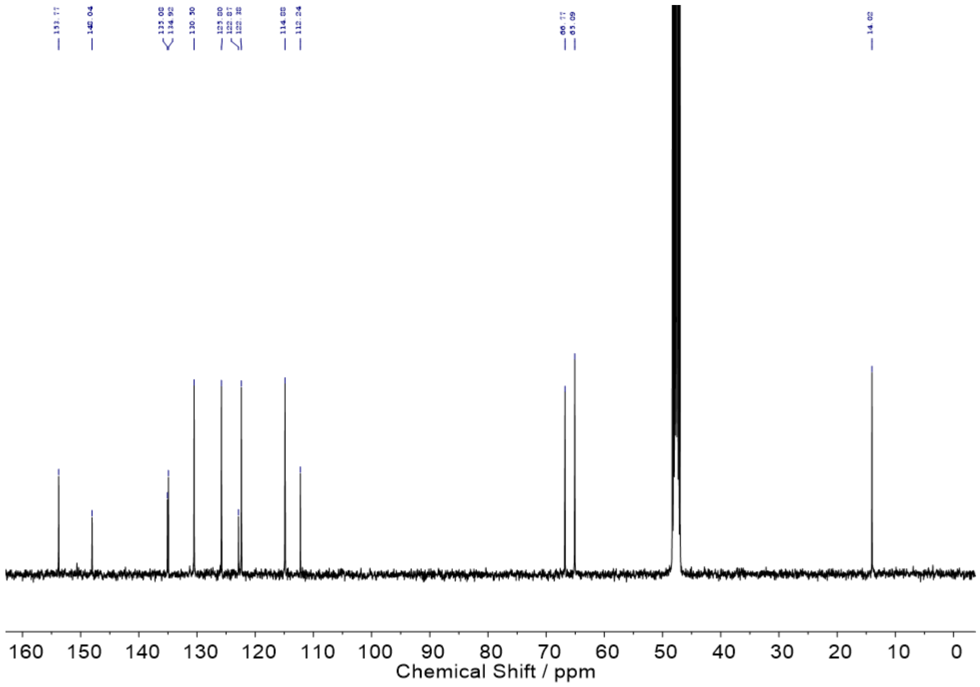

Supplementary Fig. 38. ^{13}C NMR spectrum of *O*-CL097.

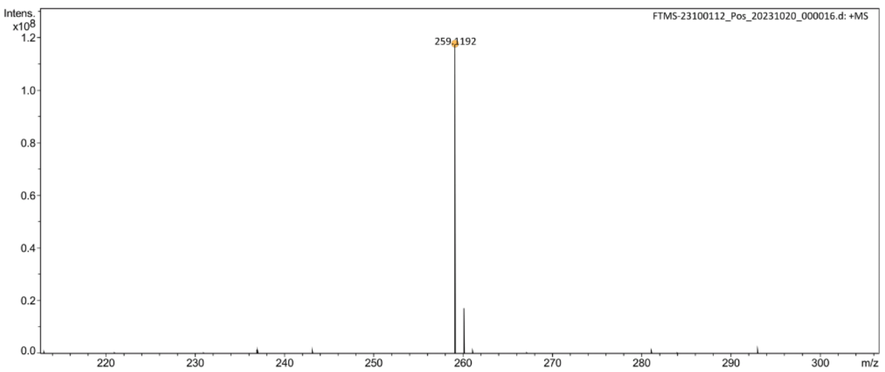

Meas. m/z	#	Ion Formula	Score	m/z	err [ppm]	Mean err [ppm]	mSigma	rdb	e⁻ Conf	N-Rule
259.1192	1	C13H15N4O2	100.00	259.1190	-1.0	-1.0	0.8	9.0	even	ok

Supplementary Fig. 39. High-resolution mass spectrum of *O*-CL097.

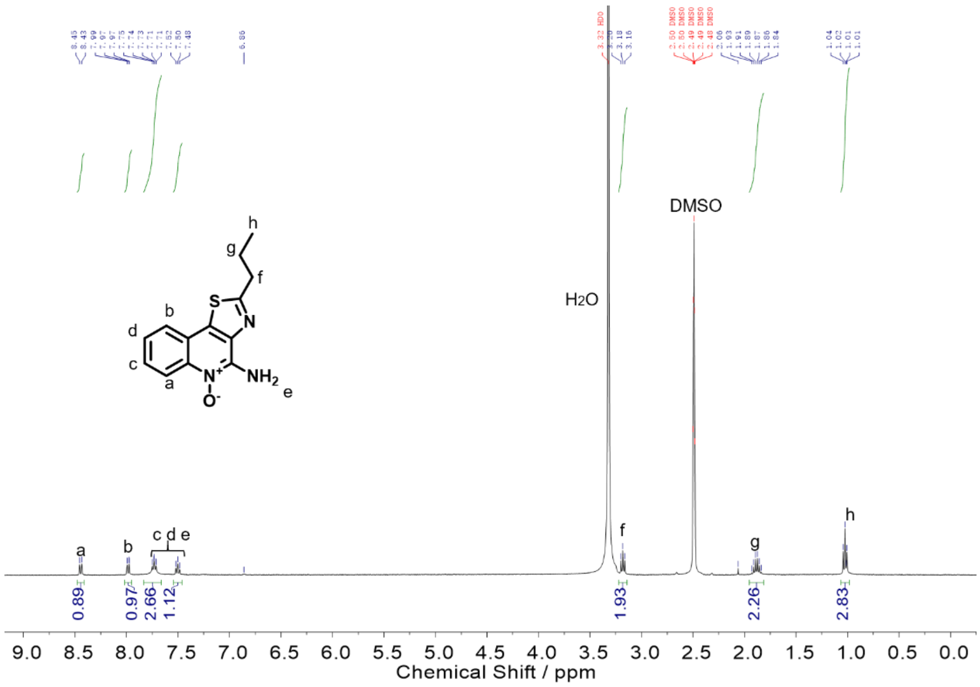

Supplementary Fig. 40. ^{1}H NMR spectrum of *O*-CL075.

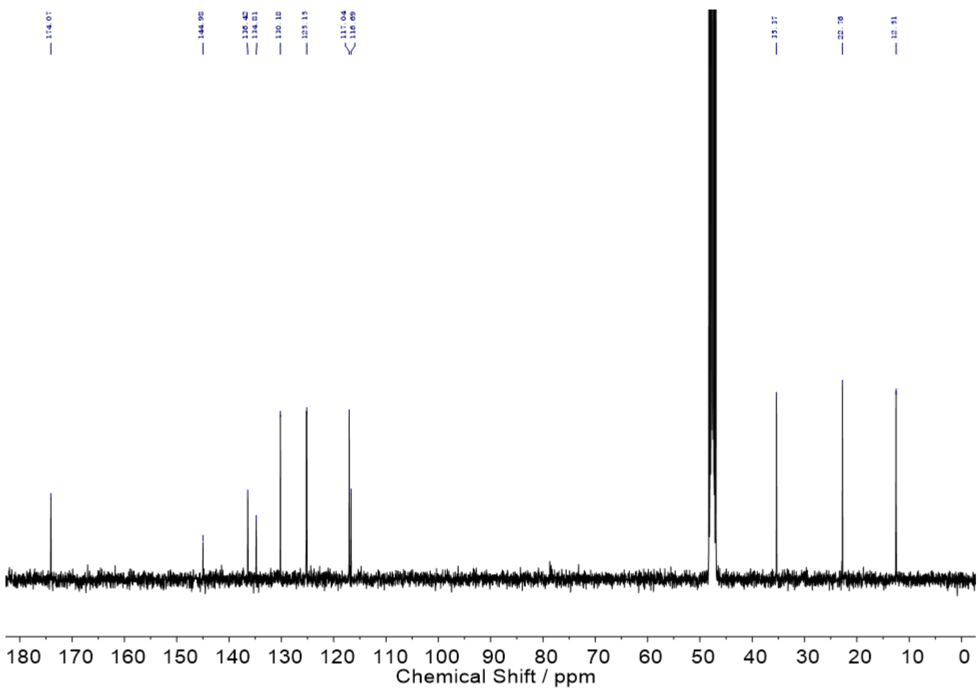

Supplementary Fig. 41. ^{13}C NMR spectrum of *O*-CL075.

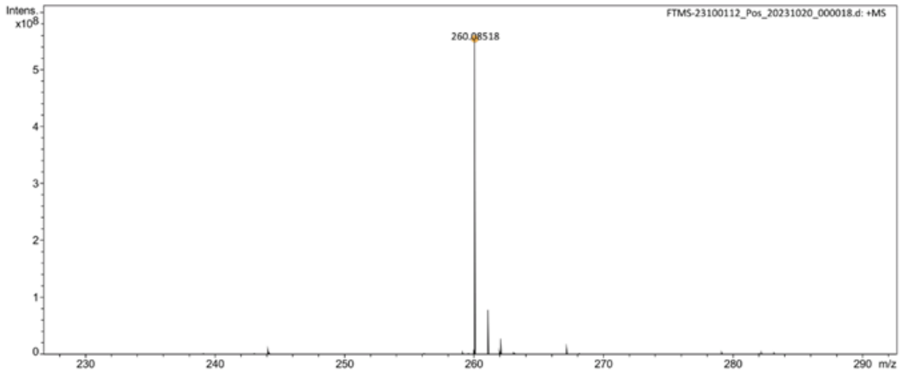

Meas. m/z	#	Ion Formula	Score	m/z	err [ppm]	Mean err [ppm]	mSigma	rdb	e⁻ Conf	N-Rule
260.08518	1	C13H14N3OS	100.00	260.08521	0.11	0.91	4.0	11.0	even	ok

Supplementary Fig. 42. High-resolution mass spectrum of *O*-CL075.

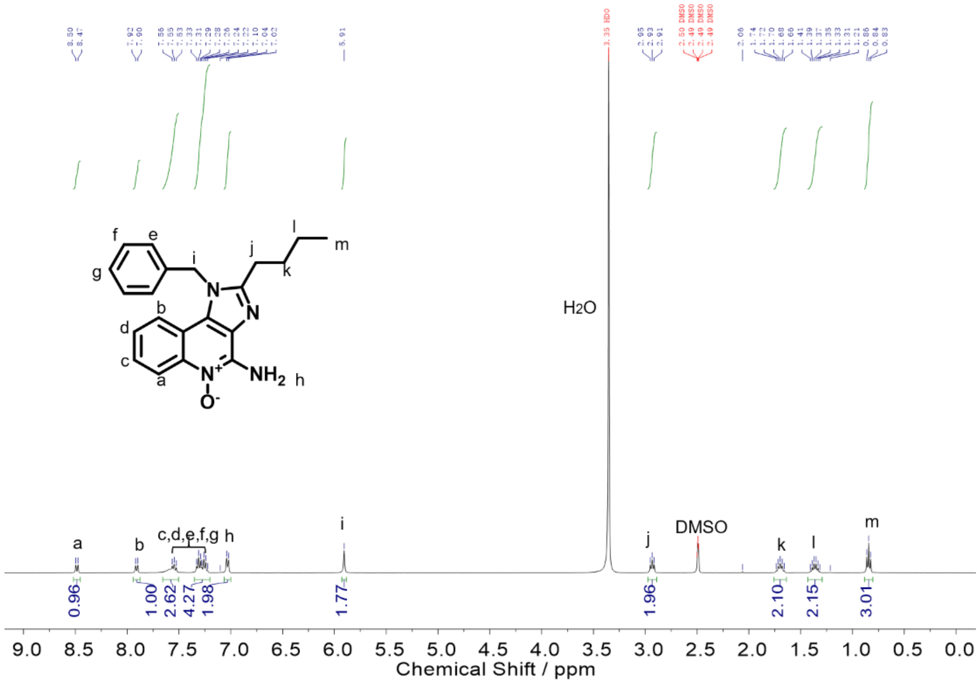

Supplementary Fig. 43. ^{1}H NMR spectrum of *O*-BBIQ.

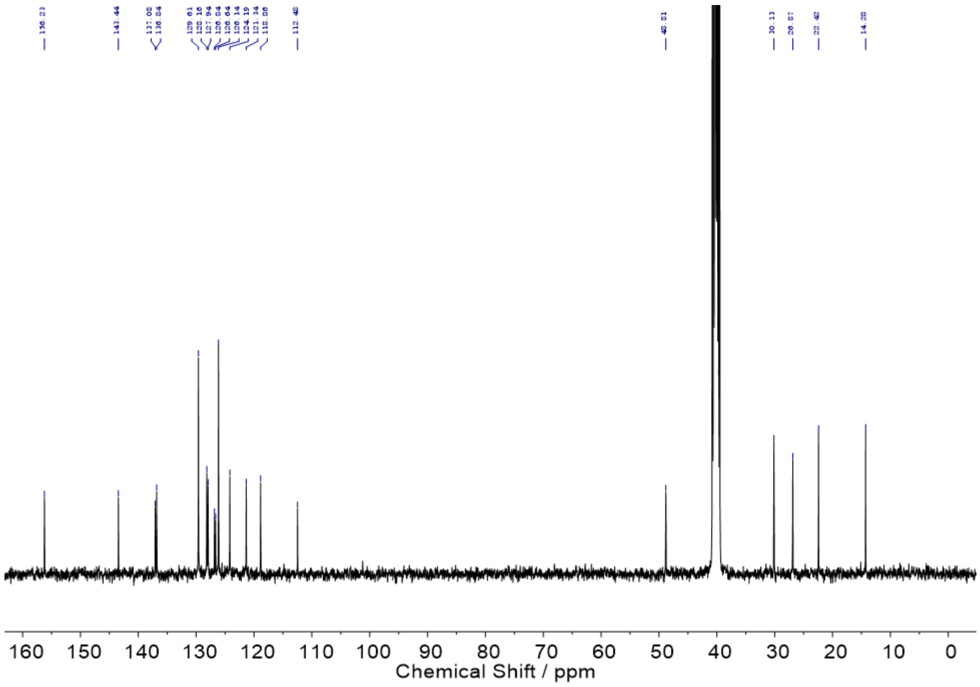

Supplementary Fig. 44. ^{13}C NMR spectrum of *O*-BBIQ.

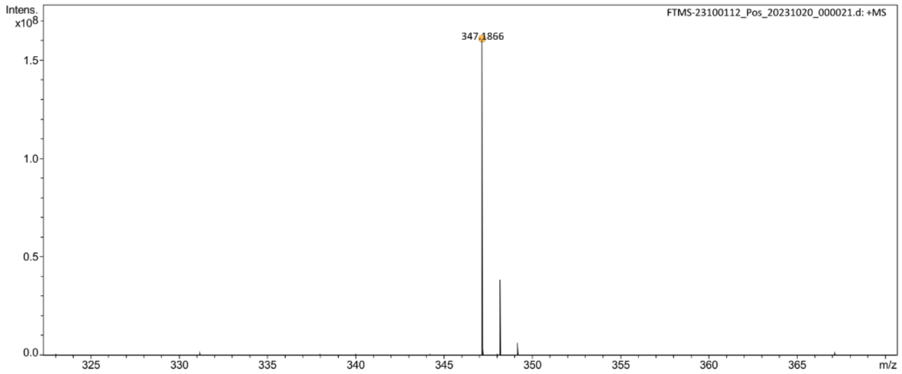

Meas. m/z	#	Ion Formula	Score	m/z	err [ppm]	Mean err [ppm]	mSigma	rdb	e⁻ Conf	N-Rule
347.1866	1	C21H23N4O	100.00	347.1866	0.0	0.0	2.4	13.0	even	ok

Supplementary Fig. 45. High-resolution mass spectrum of *O*-BBIQ.

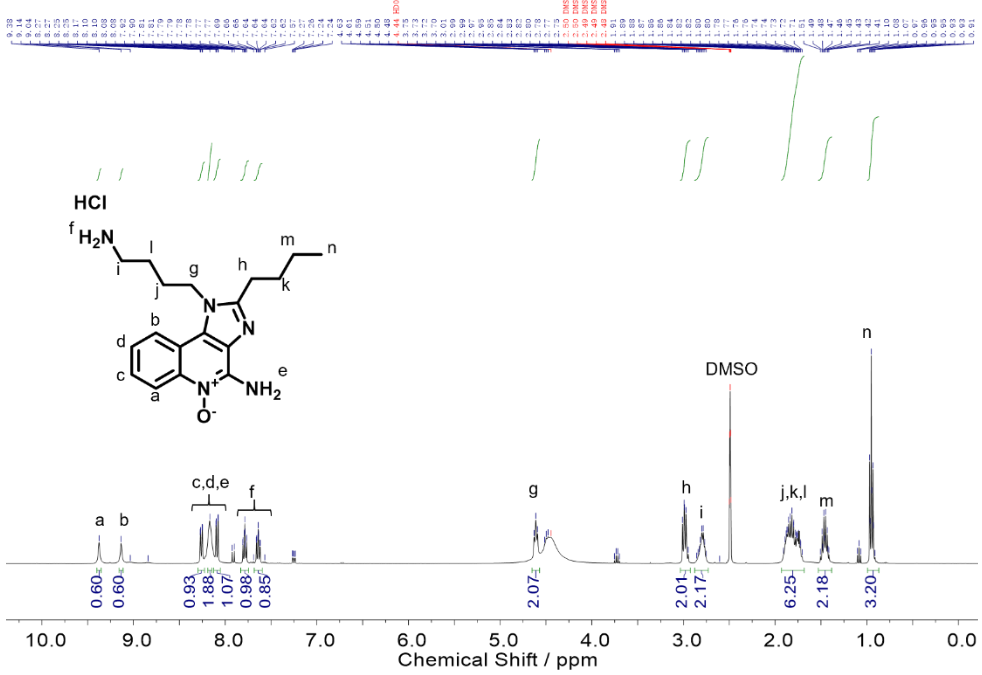

Supplementary Fig. 46. ^{1}H NMR spectrum of *O*-T785.

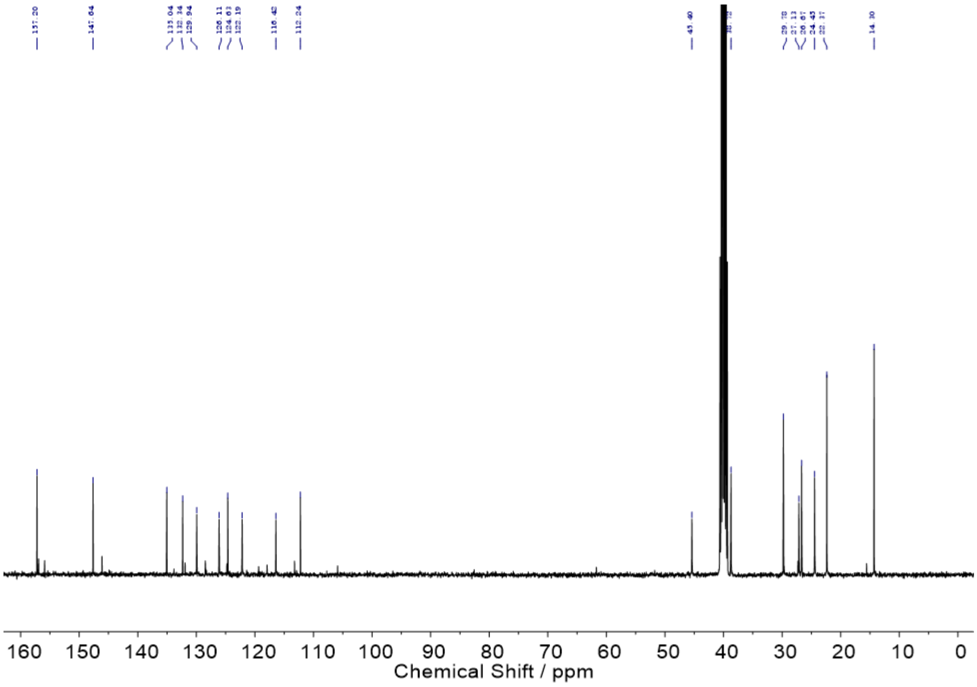

Supplementary Fig. 47. ^{13}C NMR spectrum of *O*-T785.

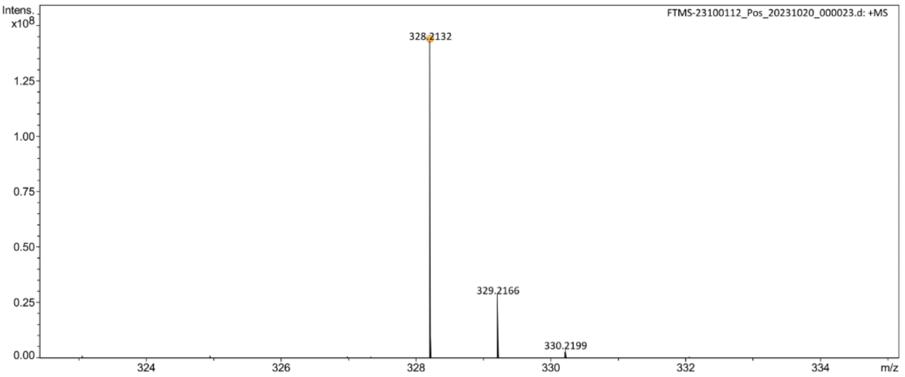

Meas. m/z	#	Ion Formula	Score	m/z	err [ppm]	Mean err [ppm]	mSigma	rdb	e⁻ Conf	N-Rule
328.2132	1	C18H26N5O	100.00	328.2132	0.0	-0.0	0.9	9.0	even	ok

Supplementary Fig. 48. High-resolution mass spectrum of *O*-T785.

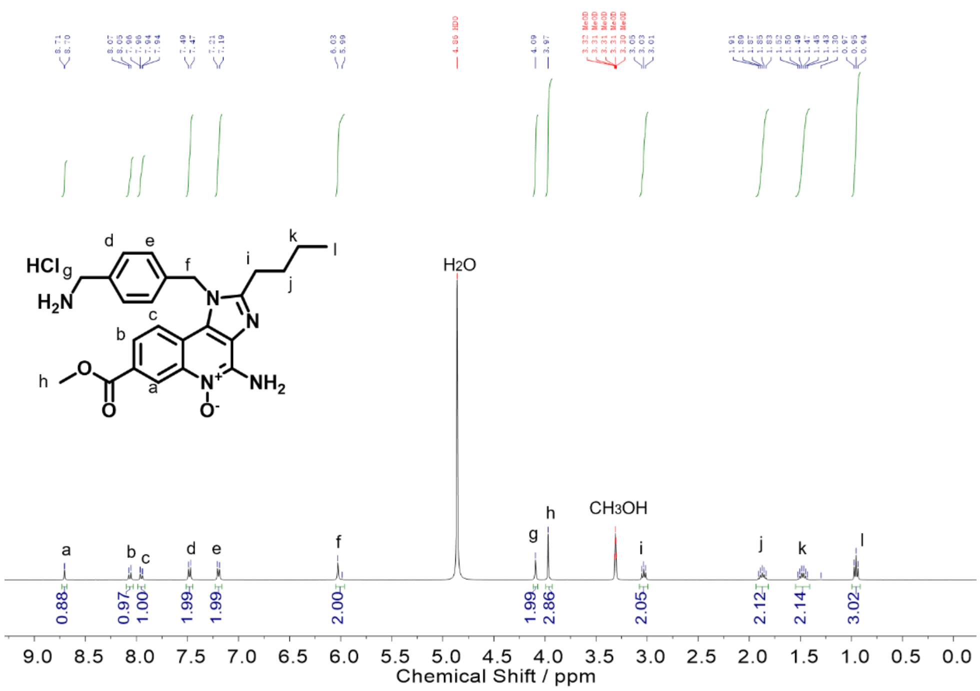

Supplementary Fig. 49. ^{1}H NMR spectrum of *O*-IMQ1.

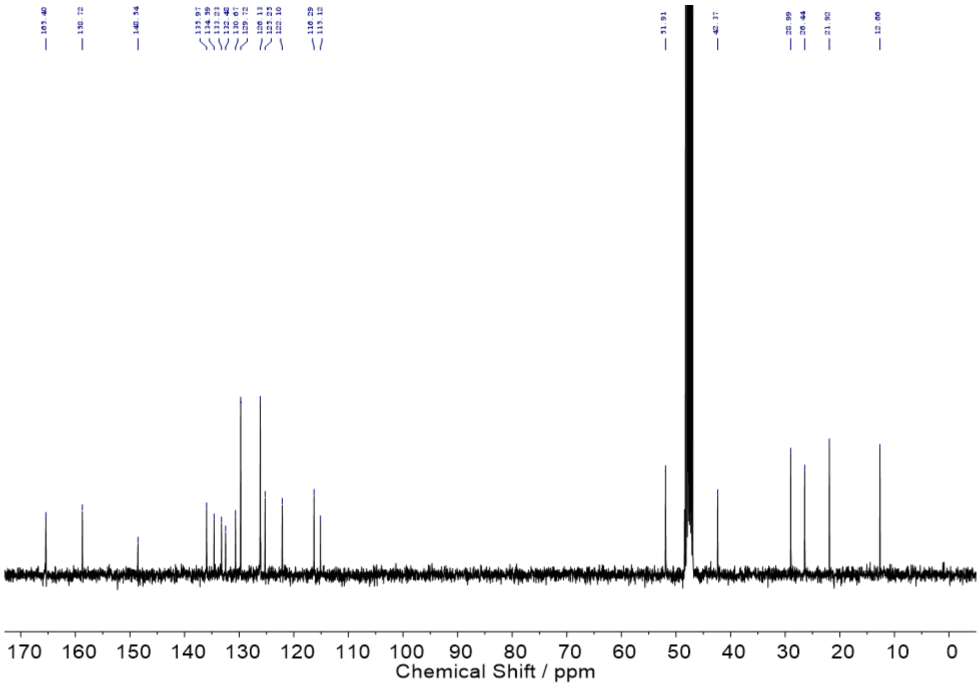

Supplementary Fig. 50. ^{13}C NMR spectrum of *O*-IMQ1.

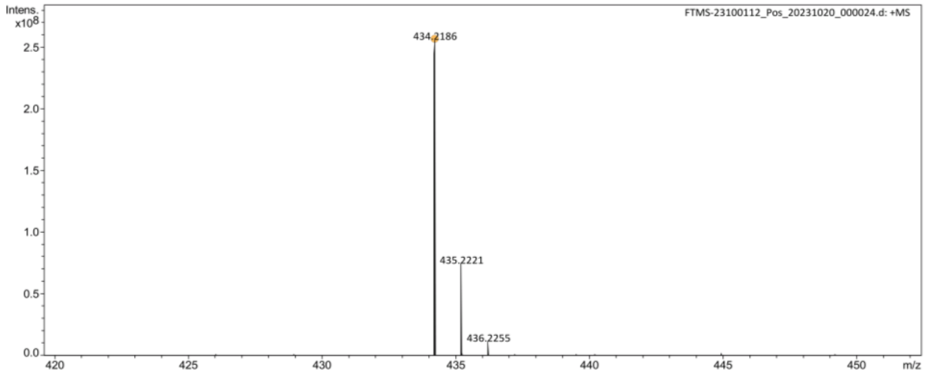

Meas. m/z	#	Ion Formula	Score	m/z	err [ppm]	Mean err [ppm]	mSigma	rdb	e⁻ Conf	N-Rule
434.2186	1	C24H28N5O3	100.00	434.2187	0.2	0.1	9.5	14.0	even	ok

Supplementary Fig. 51. High-resolution mass spectrum of *O*-IMQ1.

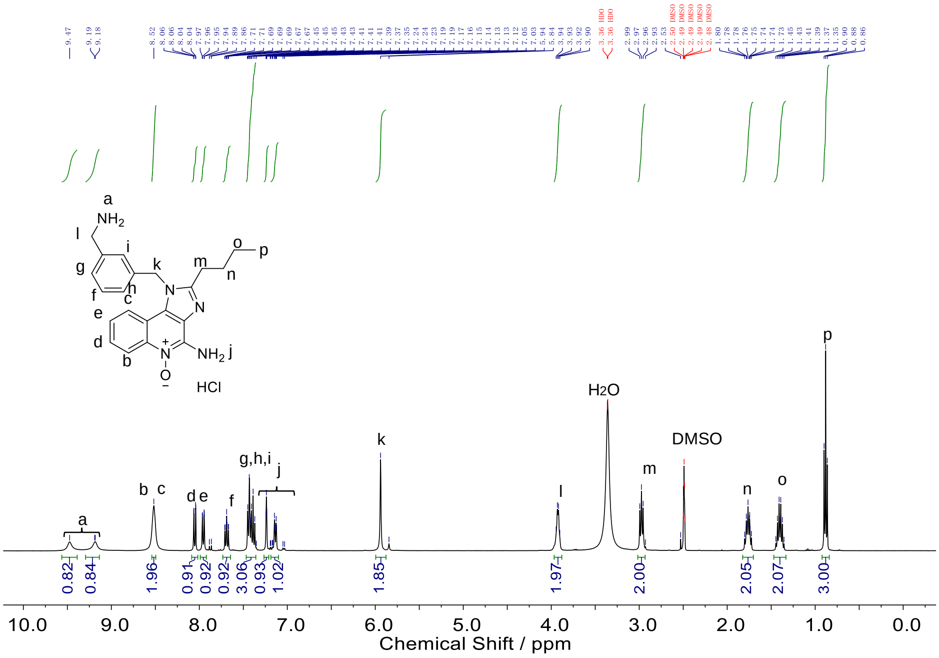

Supplementary Fig. 52. ^{1}H NMR spectrum of *O*-IMQ2.

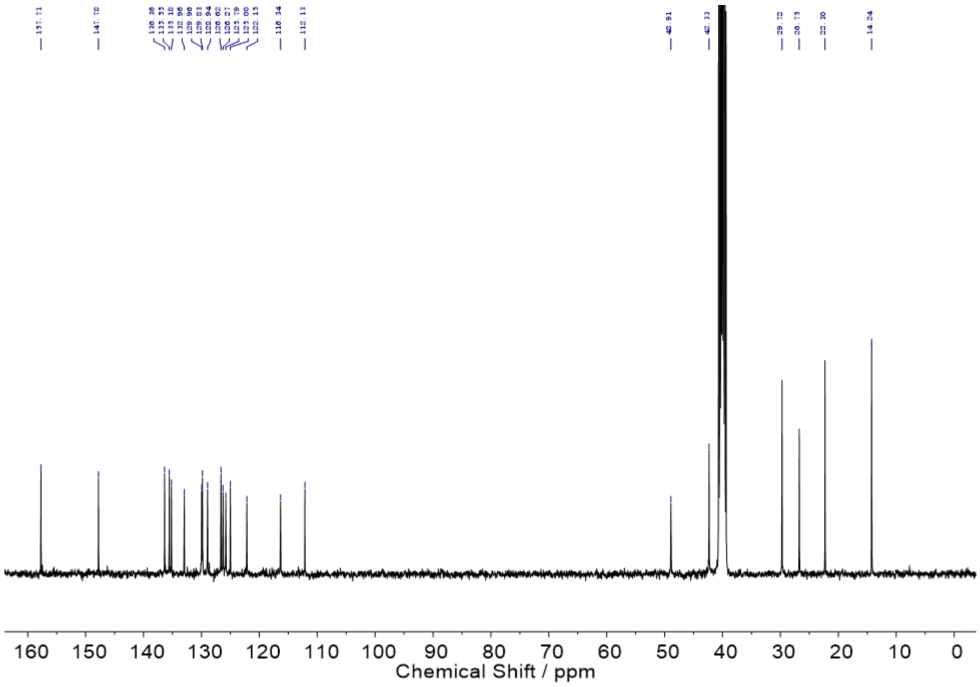

Supplementary Fig. 53. ^{13}C NMR spectrum of *O*-IMQ2.

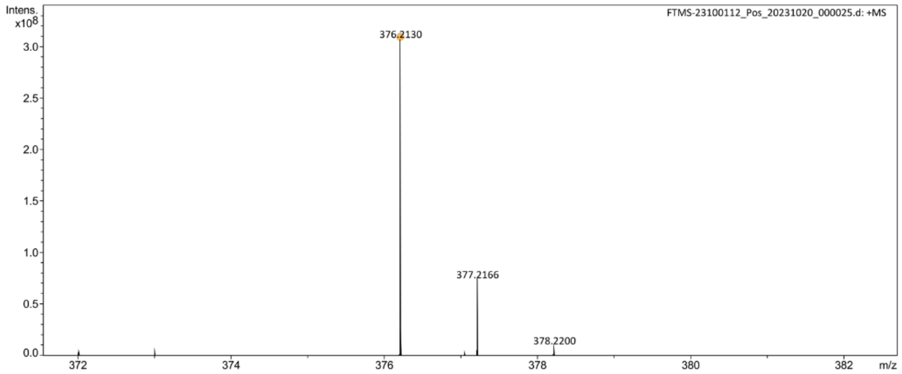

Meas. m/z	#	Ion Formula	Score	m/z	err [ppm]	Mean err [ppm]	mSigma	rdb	e⁻ Conf	N-Rule
376.2130	1	C22H26N5O	100.00	376.2132	0.4	-0.0	1.2	13.0	even	ok

Supplementary Fig. 54. High-resolution mass spectrum of *O*-IMQ2.

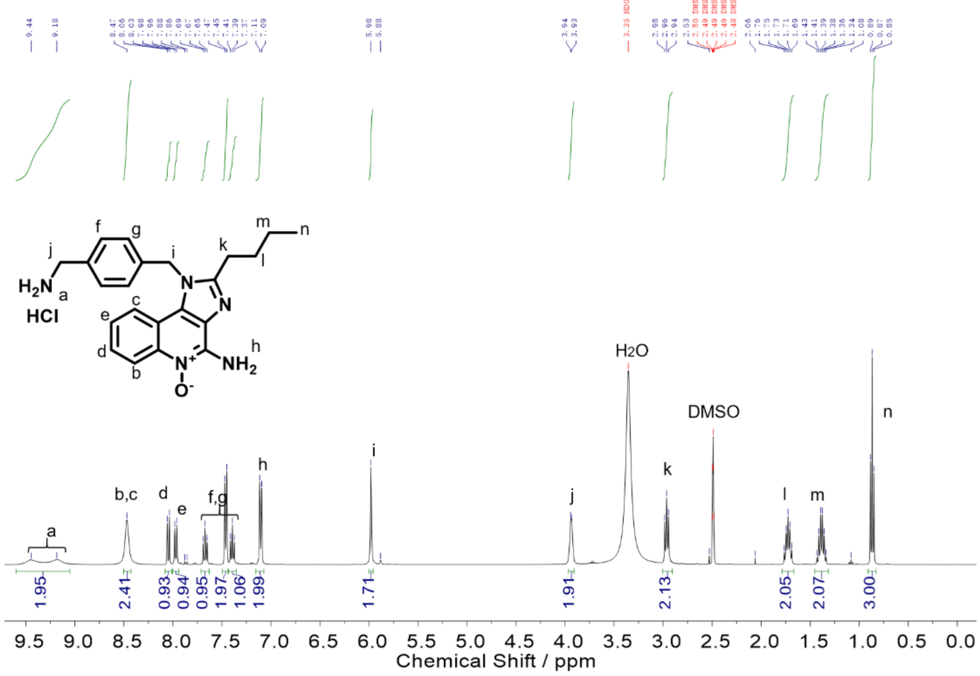

Supplementary Fig. 55. ^{1}H NMR spectrum of *O*-IMQ3.

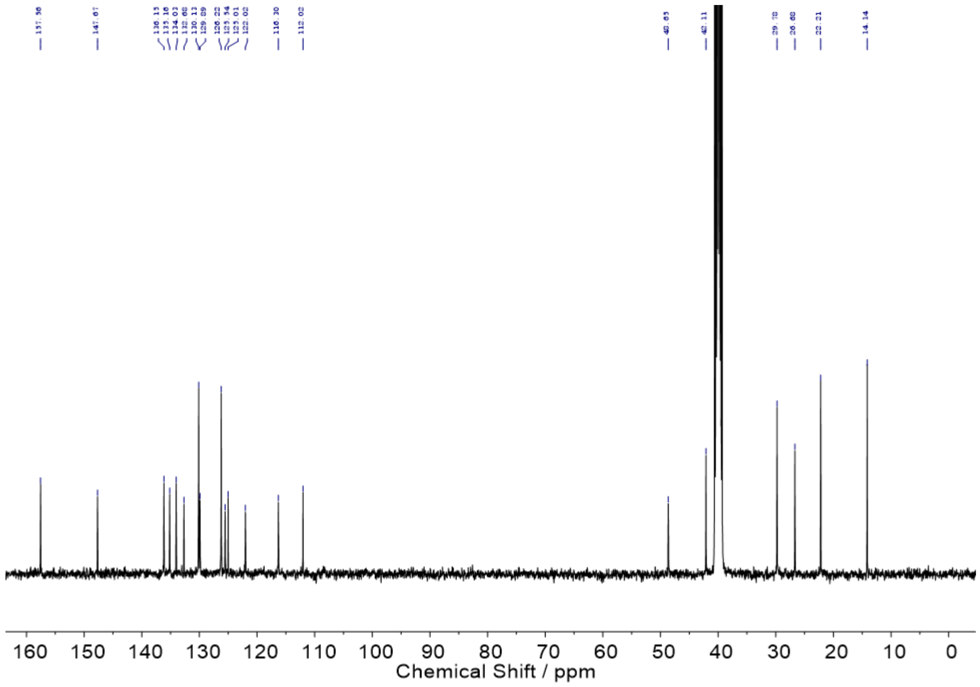

Supplementary Fig. 56. ^{13}C NMR spectrum of *O*-IMQ3.

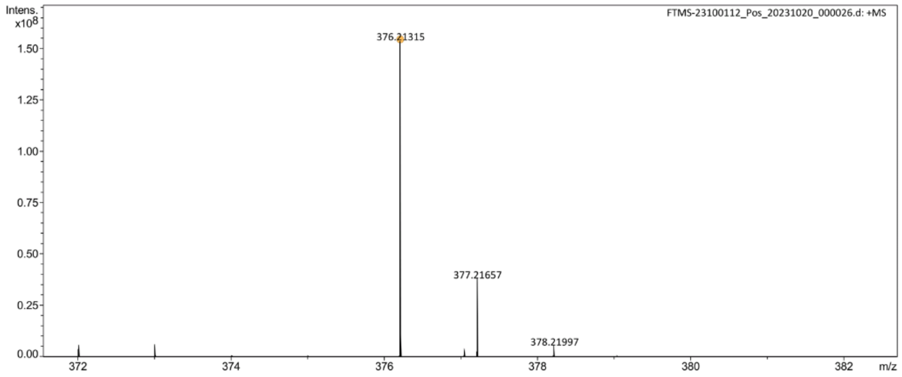

Meas. m/z	#	Ion Formula	Score	m/z	err [ppm]	Mean err [ppm]	mSigma	rdb	e⁻ Conf	N-Rule
376.21315	1	C22H26N5O	100.00	376.21319	0.10	-0.04	1.4	13.0	even	ok

Supplementary Fig. 57. High-resolution mass spectrum of *O*-IMQ3.

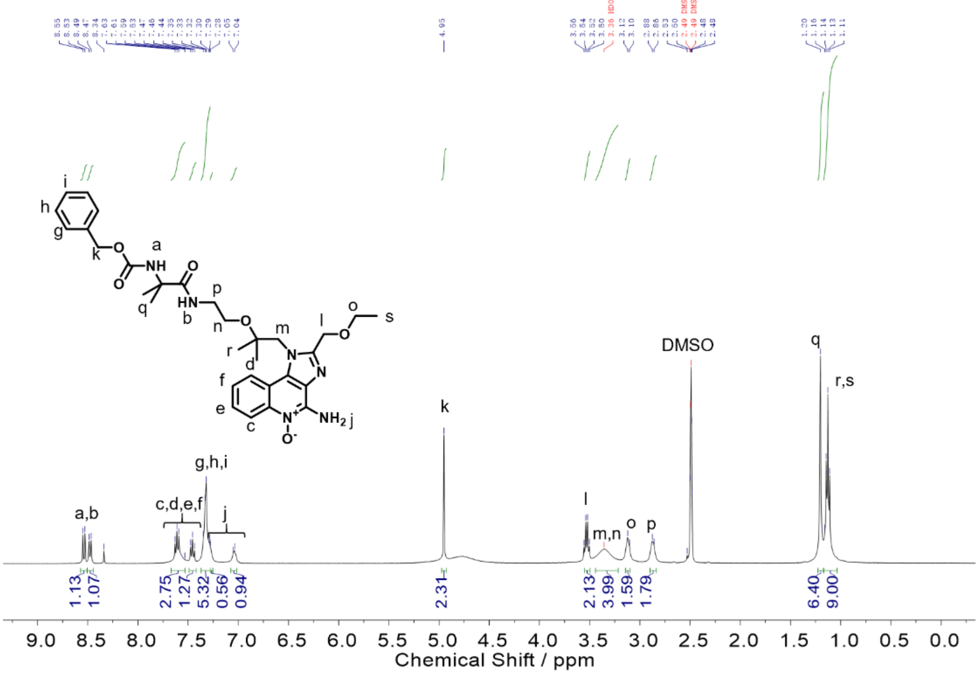

Supplementary Fig. 58. ^{1}H NMR spectrum of *O*-IMQ4.

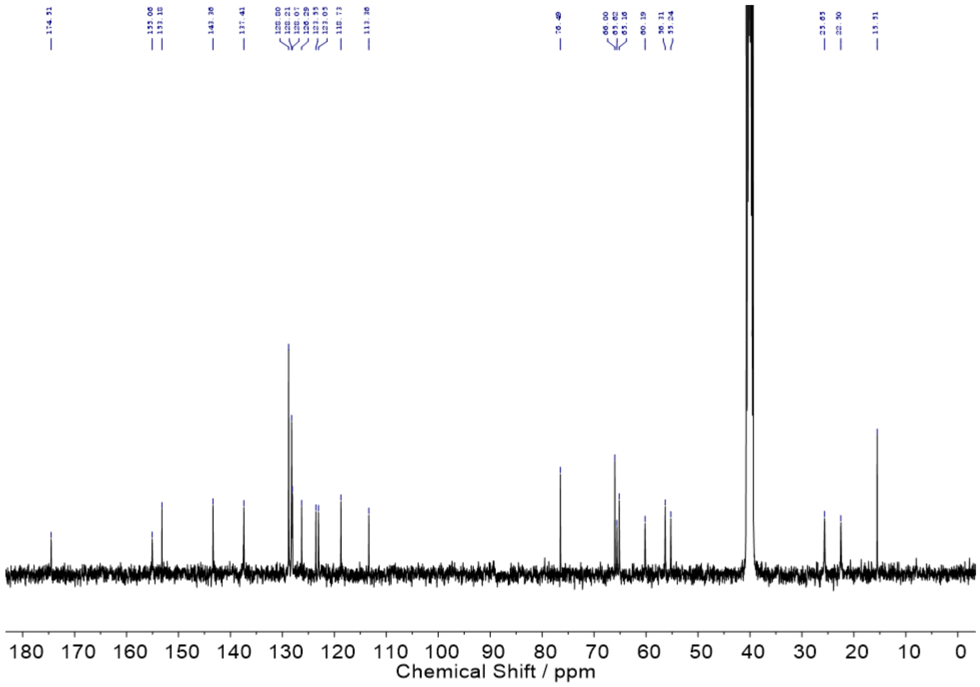

Supplementary Fig. 59. ^{13}C NMR spectrum of *O*-IMQ4.

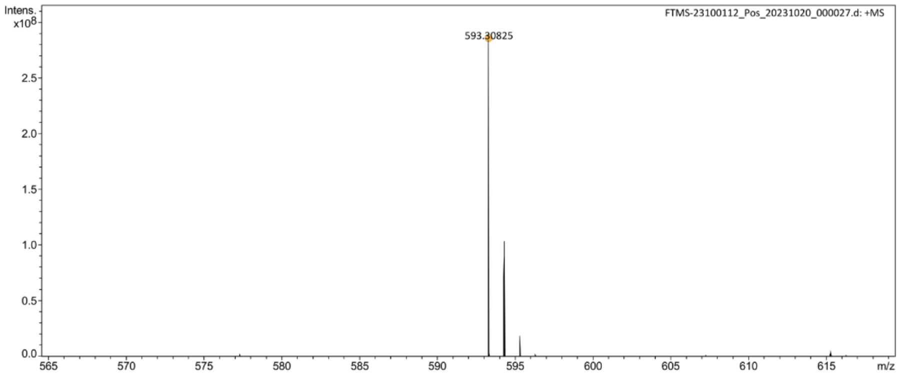

Meas. m/z	#	Ion Formula	Score	m/z	err [ppm]	Mean err [ppm]	mSigma	rdb	e⁻ Conf	N-Rule
593.30825	1	C31H41N6O6	100.00	593.30821	-0.07	-1.33	11.4	15.0	even	ok

Supplementary Fig. 60. High-resolution mass spectrum of *O*-IMQ4.

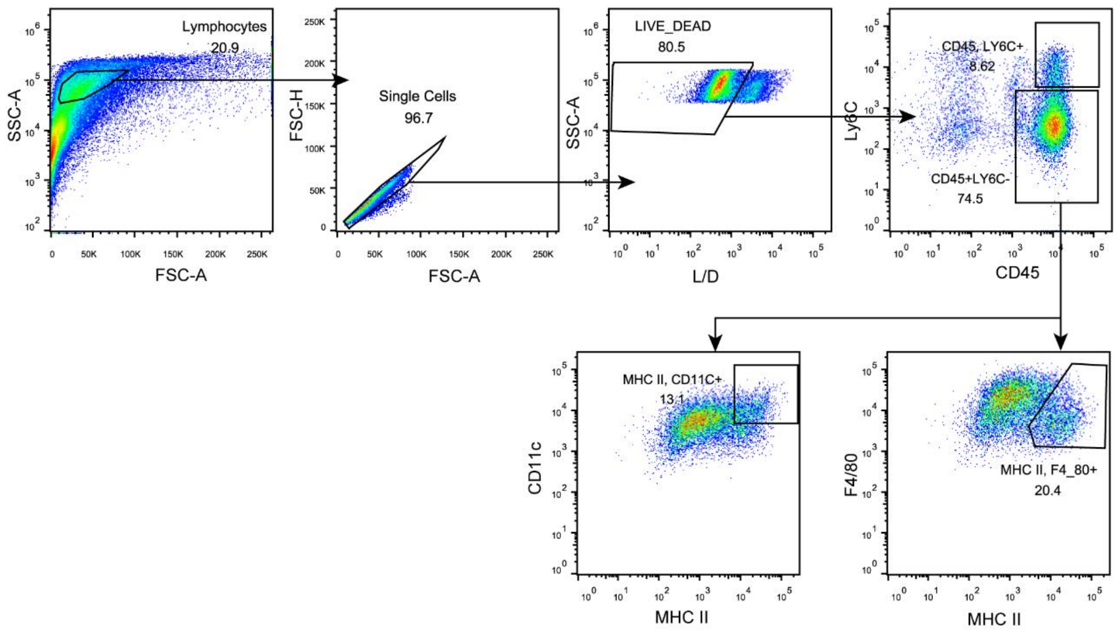

Supplementary Fig. 61. Gating strategies used for cell sorting. Gating strategy to sort CD45$^+$MHC-II$^+$CD11c$^+$ TIDCs, CD45$^+$MHC-II$^+$f4/80$^+$ TAMs, and CD45$^+$Ly6C$^+$ MDSCs for *in vitro* cultures presented on Fig. 4b-d.

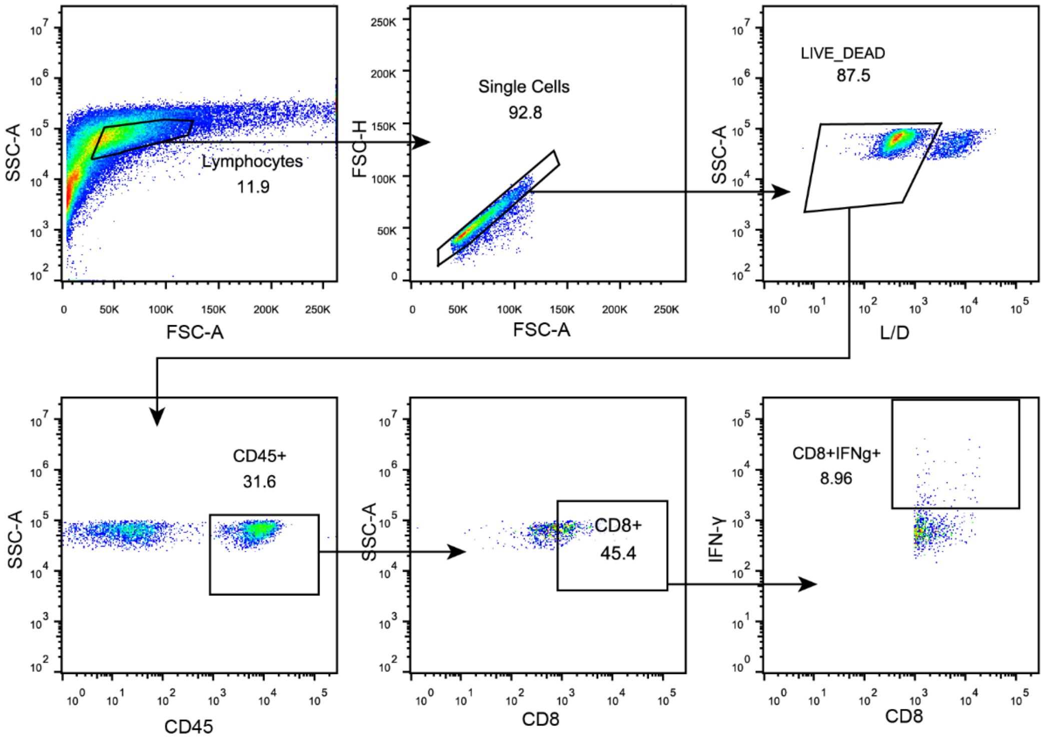

Supplementary Fig. 62. Gating strategies used for cell sorting. Gating strategy to sort $CD45^{+}CD8^{+}$ T cells for in vitro cultures presented on Fig. 4e-h.

Table 1. The effective permeability coefficients (Pe) of R848 and *O*-R848.

c	Pe (10^{-6} cm/s)
R848	14.62 ± 1.31
O-R848	0.43 ± 0.09

The Pe value was determined by parallel artificial membrane permeability assay (PAMPA), *n* = 3. Source data are provided as a Source Data file.

Table 2. Calculated Gibbs free energies of oxygen-engineered agonists and their anions

	O(Hartree)[a]	*[O]⁻(Hartree)*[b]	ΔG *(kJ/mol)*[c]
***O*-852A**	-1519.961	-1520.037	-199.538
***O*-BBIQ**	-1276.464	-1276.548	-220.247
***O*-CL075**	-1180.210	-1180.299	-233.669
***O*-CL097**	-873.295	-873.372	-202.163
***O*-Gar**	-1046.538	-1046.614	-199.538
***O*-IMQ1**	-1565.378	-1565.453	-197.235
***O*-IMQ2**	-1452.871	-1452.954	-217.634
***O*-IMQ3**	-1473.166	-1473.243	-201.037
***O*-IMQ4**	-1974.994	-1975.074	-209.543
***O*-R837**	-1026.503	-1026.577	-193.169
***O*-R848**	-1105.717	-1105.798	-212.665
***O*-T785**	-1356.063	-1356.143	-209.375

[a]The electronic energy of oxygen-engineered agonists calculated by B3-LYP in the water solvent. [b]The electronic energy of oxygen-engineered agonists anion calculated by B3-LYP in the water solvent. [c]The electron affinity of oxygen-engineered agonists (ΔG). Source data are provided as a Source Data file.

Table 3. The catalogue numbers of the antibodies used in this article

Antibody used in this article	Dilutions	Catalogue numbers	Clone numbers
anti-mouse CD8α-APC	≤0.125 µg per million cells in 100 µL volume	Biolegend, #126614	YTS156.7.7
anti-mouse CD3-BV421	≤0.25 µg per million cells in 100 µL volume	Biolegend, #100228	17A2
anti-CD16/32 antibody	≤1.0 µg per million cells in 100 µL volume	Biolegend, #101301	93
anti-mouse IFN-γ	≤1.0 µg per million cells in 100 µL volume	Biolegend, #505809	XMG1.2
anti-mouse Ki67	≤0.5 µg per million cells in 100 µL volume	Biolegend, #652401	16A8
