# Supplementary material for: Single atom engineering for radiotherapy-activated immune agonist prodrugs
Source: Nat Commun. 2025 Jul 1;16:6021. doi: 10.1038/s41467-025-60768-4 (PMC12218326; doi:10.1038/s41467-025-60768-4)
Supplement: Supplementary file 3 — Reporting Summary [file 41467_2025_60768_MOESM3_ESM.pdf]

nature portfolio

Corresponding author(s): Zhibo Liu, Yang-Xin Fu

Last updated by author(s): Feb 18, 2025

Reporting Summary

Nature Portfolio wishes to improve the reproducibility of the work that we publish. This form provides structure for consistency and transparency in reporting. For further information on Nature Portfolio policies, see our Editorial Policies and the Editorial Policy Checklist.

Statistics

For all statistical analyses, confirm that the following items are present in the figure legend, table legend, main text, or Methods section.

n/a	Confirmed	
☐	☒	The exact sample size (*n*) for each experimental group/condition, given as a discrete number and unit of measurement
☐	☒	A statement on whether measurements were taken from distinct samples or whether the same sample was measured repeatedly
☐	☒	The statistical test(s) used AND whether they are one- or two-sided<br>*Only common tests should be described solely by name; describe more complex techniques in the Methods section.*
☒	☐	A description of all covariates tested
☒	☐	A description of any assumptions or corrections, such as tests of normality and adjustment for multiple comparisons
☐	☒	A full description of the statistical parameters including central tendency (e.g. means) or other basic estimates (e.g. regression coefficient) AND variation (e.g. standard deviation) or associated estimates of uncertainty (e.g. confidence intervals)
☐	☒	For null hypothesis testing, the test statistic (e.g. *F*, *t*, *r*) with confidence intervals, effect sizes, degrees of freedom and *P* value noted<br>*Give P values as exact values whenever suitable.*
☒	☐	For Bayesian analysis, information on the choice of priors and Markov chain Monte Carlo settings
☐	☒	For hierarchical and complex designs, identification of the appropriate level for tests and full reporting of outcomes
☒	☐	Estimates of effect sizes (e.g. Cohen's *d*, Pearson's *r*), indicating how they were calculated

Our web collection on statistics for biologists contains articles on many of the points above.

Software and code

Policy information about availability of computer code

Data collection	Characterization of SAE-RAP: X-ray irradiation was delivered by an X-ray generator (RS2000 Pro 225, 225 kV, 17.7 mA, Rad Source Technologies, Inc.). Nuclear magnetic resonance (NMR) spectra were recorded on Bruker AVANCE 400 MHz spectrometer. Signals are presented as parts per million (ppm), and multiplicity is presented as single (s), broad (b), doublet (d), triplet (t), quartet (q), or multiplet (m). Ultra-performance liquid chromatography (UPLC-MS) was performed on ACQUITY UPLC H-Class PLUS instrument equipped with Waters PDA eλ Detector and a Waters SQ Detector 2. prep-HPLC (neutral condition, column: Waters Xbridge Prep OBD C18). High-resolution mass spectroscopy was performed on a Bruker Fourier Transform Ion Cyclotron Resonance Mass Spectrometer. Fluorescence spectra were measured on an F-7000 spectrophotometer (Hitachi, Japan). Flow cytometry was performed on FACS Fortessa(BD Biosciences)Clinical. The absorbance of cells was measured on TECAN Infinite E Plex. The residue analysis, molecule docking, and interaction analysis were performed with Molecular Operating Environment (MOE, Canada), GOLD (CCDC, UK) and Discovery Studio Visualizer (BIOVIA), respectively. The molecular dynamics (MD) was performed with Assisted Model Building with Energy Refinement (AMBER 20).H&E staining: 3Dhistech(3DHISTECH)
Data analysis	NMR data analysis: MestReNova; UPLC data analysis: MassLynx v4.1; 3D structures modeling and MD simulation: VMD v19.3; Statistical analysis: GraphPad Prism v8, and Origin 2023b; Flow cytometry analysis: FlowJo v10.8.1; H&E staining: CaseViewer v2.4

For manuscripts utilizing custom algorithms or software that are central to the research but not yet described in published literature, software must be made available to editors and reviewers. We strongly encourage code deposition in a community repository (e.g. GitHub). See the Nature Portfolio guidelines for submitting code & software for further information.

Data

Policy information about availability of data

All manuscripts must include a data availability statement. This statement should provide the following information, where applicable:

- Accession codes, unique identifiers, or web links for publicly available datasets
- A description of any restrictions on data availability
- For clinical datasets or third party data, please ensure that the statement adheres to our policy

All data supporting the results of this study are available within the paper and its Supplementary Information. Source data are provided in this paper.

Research involving human participants, their data, or biological material

Policy information about studies with human participants or human data. See also policy information about sex, gender (identity/presentation), and sexual orientation and race, ethnicity and racism.

Reporting on sex and gender	*Use the terms sex (biological attribute) and gender (shaped by social and cultural circumstances) carefully in order to avoid confusing both terms. Indicate if findings apply to only one sex or gender; describe whether sex and gender were considered in study design; whether sex and/or gender was determined based on self-reporting or assigned and methods used. Provide in the source data disaggregated sex and gender data, where this information has been collected, and if consent has been obtained for sharing of individual-level data; provide overall numbers in this Reporting Summary. Please state if this information has not been collected. Report sex- and gender-based analyses where performed, justify reasons for lack of sex- and gender-based analysis.*
Reporting on race, ethnicity, or other socially relevant groupings	*Please specify the socially constructed or socially relevant categorization variable(s) used in your manuscript and explain why they were used. Please note that such variables should not be used as proxies for other socially constructed/relevant variables (for example, race or ethnicity should not be used as a proxy for socioeconomic status). Provide clear definitions of the relevant terms used, how they were provided (by the participants/respondents, the researchers, or third parties), and the method(s) used to classify people into the different categories (e.g. self-report, census or administrative data, social media data, etc.) Please provide details about how you controlled for confounding variables in your analyses.*
Population characteristics	*Describe the covariate-relevant population characteristics of the human research participants (e.g. age, genotypic information, past and current diagnosis and treatment categories). If you filled out the behavioural & social sciences study design questions and have nothing to add here, write "See above."*
Recruitment	*Describe how participants were recruited. Outline any potential self-selection bias or other biases that may be present and how these are likely to impact results.*
Ethics oversight	*Identify the organization(s) that approved the study protocol.*

Note that full information on the approval of the study protocol must also be provided in the manuscript.

Field-specific reporting

Please select the one below that is the best fit for your research. If you are not sure, read the appropriate sections before making your selection.

☒ Life sciences ☐ Behavioural & social sciences ☐ Ecological, evolutionary & environmental sciences

For a reference copy of the document with all sections, see nature.com/documents/nr-reporting-summary-flat.pdf

Life sciences study design

All studies must disclose on these points even when the disclosure is negative.

Sample size	Exact sample size is stated in the figure legend. in the animal experiments to detect tumour growth, for individual analyses n is 6 in each group. For all other experiment, for individual analyses n is 3, 5,or 6, selected on the basis that the variability between estimates is sufficiently small to provide significant differences between test samples in these studies.
Data exclusions	No data were excluded from the analyses
Replication	All results were repeated at least three times independently with similar results.
Randomization	Randomization was applied following these two criteria: the probability of assignment to any of the experimental groups is equal for each subject and the assignment of one subject to a group does not affect the assignment of any other subject to that same group
Blinding	Proper blinding was applied during the data collection and analysis.

Reporting for specific materials, systems and methods

We require information from authors about some types of materials, experimental systems and methods used in many studies. Here, indicate whether each material, system or method listed is relevant to your study. If you are not sure if a list item applies to your research, read the appropriate section before selecting a response.

Materials & experimental systems

n/a	Involved in the study
☐	☒ Antibodies
☐	☒ Eukaryotic cell lines
☒	☐ Palaeontology and archaeology
☐	☒ Animals and other organisms
☒	☐ Clinical data
☒	☐ Dual use research of concern
☒	☐ Plants

Methods

n/a	Involved in the study
☒	☐ ChIP-seq
☐	☒ Flow cytometry
☒	☐ MRI-based neuroimaging

Antibodies

Antibodies used	anti-mouse CD8α-APC (≤0.125 µg per million cells in 100 µl volume)(Biolegend, #126614,clone YTS156.7.7), anti-mouse CD3-BV421(≤ 0.25 µg per million cells in 100 µl volume)(Biolegend, #100228,clone 17A2), anti-CD16/32 antibody(≤ 1.0 µg per million cells in 100 µl volume)(Biolegend, #101301,clone 93), APC anti-mouse IFN-γ Antibody(≤1.0 µg per million cells in 100 µl volume) (BioLegend, 505809, clone XMG1.2), anti-mouse Ki67(≤ 0.5 µg per million cells in 100 µl volume)(Biolegend, #652401,clone 16A8)
Validation	anti-mouse CD8α-APC (Biolegend, #126614): Rat anti-mouse antibody, Flow cytometry analysis; anti-mouse CD3-BV421(Biolegend, #100228): Rat anti-mouse antibody, Flow cytometry analysis; anti-CD16/32 antibody(Biolegend, #101301): Rat anti-mouse antibody, Flow cytometry analysis; anti-mouse IFN-γ(Biolegend, #517902): Rat anti-mouse antibody, Flow cytometry analysis; anti-mouse Ki67(Biolegend, #151202): Rat anti-mouse antibody, Flow cytometry analysis.

Eukaryotic cell lines

Policy information about cell lines and Sex and Gender in Research

Cell line source(s)	The MC38 cell line(#SNL-505) were purchased from SUNNCELL. The B16(#CL-0029), 4T1(#CL-0007), A549(#CL-0016), CT26(#CL-0071), HEK 293(#CL-0001)and RAW264.7 (#CL-0190)cell lines were purchased from Procell. RAW-Blue reporter cells(RAW-Blue™ Cells) were purchased from Invivogen.
Authentication	Identity of the cell lines were frequently checked by their morphological features but have not been authenticated by shorttandem repeat (STR) profiling.
Mycoplasma contamination	All cell lines tested negative for mycoplasma contamination.
Commonly misidentified lines (See ICLAC register)	No commonly misidentified cell line was used in the study.

Animals and other research organisms

Policy information about studies involving animals; ARRIVE guidelines recommended for reporting animal research, and Sex and Gender in Research

Laboratory animals	6-8-week-old female C57BL/6J (#213)and BALB/c (#211)mice were obtained from Beijing Vital River Laboratory Animal Technology Co, ltd. and maintained under specific pathogen-free facility (SPF) conditions with a 12 light/12 dark cycle, and free access to food and water, Mice were housed under temperature of 24±2?,and humidity of 50±10%.
Wild animals	The study did not involve wild animals
Reporting on sex	The mice used in this experiment are all female(Considering that female mice exhibit lower aggression when living in groups, which are more suitable for group breeding with reduced management difficulty)
Field-collected samples	The study did not involve samples collected from the field
Ethics oversight	All animal care and experimental procedure were performed by following the animal protocols(IACUC ID: CCME-LiuZB-2) approved by the ethics committee of Peking University

Note that full information on the approval of the study protocol must also be provided in the manuscript.

Plants

Seed stocks	*Report on the source of all seed stocks or other plant material used. If applicable, state the seed stock centre and catalogue number. If plant specimens were collected from the field, describe the collection location, date and sampling procedures.*
Novel plant genotypes	*Describe the methods by which all novel plant genotypes were produced. This includes those generated by transgenic approaches, gene editing, chemical/radiation-based mutagenesis and hybridization. For transgenic lines, describe the transformation method, the number of independent lines analyzed and the generation upon which experiments were performed. For gene-edited lines, describe the editor used, the endogenous sequence targeted for editing, the targeting guide RNA sequence (if applicable) and how the editor was applied.*
Authentication	*Describe any authentication procedures for each seed stock used or novel genotype generated. Describe any experiments used to assess the effect of a mutation and, where applicable, how potential secondary effects (e.g. second site T-DNA insertions, mosiacism, off-target gene editing) were examined.*

Flow Cytometry

Plots

Confirm that:

☒ The axis labels state the marker and fluorochrome used (e.g. CD4-FITC).

☒ The axis scales are clearly visible. Include numbers along axes only for bottom left plot of group (a 'group' is an analysis of identical markers).

☒ All plots are contour plots with outliers or pseudocolor plots.

☒ A numerical value for number of cells or percentage (with statistics) is provided.

Methodology

Sample preparation	For Flow cytometry: Tumour tissues were collected, cut into small pieces, and re-suspended in digestion buffer RPMI-1640 medium with 1 mg/mL type lV collagenase and 100 μg/mL DNase I). Tumours were digested for 45 min at 37°C and then passed through a 70-μm cell strainer to make single-cell suspensions. Single-cell suspensions were incubated with CD16/32 blocking antibody and stained with specific antibodies followed by established protocol.
Instrument	FACS Fortessa(BD Biosciences)
Software	FlowJo v10.8.1(Treestar)
Cell population abundance	When cells were sorted, the purity was confirmed by flow cytometry and in each case was above 90% purity.
Gating strategy	Gate boundaries were set according to control samples (FMO-fluorescence minus one or isotype controls).

☒ Tick this box to confirm that a figure exemplifying the gating strategy is provided in the Supplementary Information.
